# Fungi from *Anopheles darlingi* Root, 1926, larval breeding sites in the Brazilian Amazon

**DOI:** 10.1371/journal.pone.0312624

**Published:** 2024-12-05

**Authors:** Marta Rodrigues de Oliveira, Thiago Fernandes Souza, Adriano Nobre Arcos, Ricardo de Melo Katak, Sarah Raquel Silveira da Silva, Jeferson Chagas da Cruz, Gilvan Ferreira da Silva, Osvaldo Marinotti, Olle Terenius, Afonso Duarte Leão de Souza, Antonia Queiroz Lima de Souza

**Affiliations:** 1 Programa de Pós-graduação em Biodiversidade e Biotecnologia, Universidade Federal do Amazonas, Manaus, Amazonas, Brazil; 2 Department of Entomology and Acarology, School de Agricultura "Luiz de Queiroz", University of São Paulo (ESALQ/USP), Piracicaba, São Paulo, Brazil; 3 Programa de Pós-graduação de Biotecnologia, Universidade Federal do Amazonas, Manaus, Amazonas, Brazil; 4 Embrapa Amazônia Ocidental, Empresa Brasileira de Pesquisa Agropecuária (Embrapa) Manaus, Amazonas, Brazil; 5 Programa de Pós-Graduação em Ecologia e Conservação, Universidade Federal de Mato Grosso do Sul / UFMS, Campo Grande, Mato Grosso do Sul, Brazil; 6 Laboratório de Malária e Dengue, Instituto Nacional de Pesquisas da Amazônia (INPA), Manaus, Amazonas, Brazil; 7 Department of Biology, Indiana University, Bloomington, Indiana, United States of America; 8 Department of Cell and Molecular Biology, Microbiology, Uppsala University, Uppsala, Sweden; 9 Central Analítica—Centro de Apoio Multidisciplinar (CAM), Universidade Federal do Amazonas (UFAM), Manaus, Amazonas, Brazil; 10 Departamento de Química, Universidade Federal do Amazonas, Manaus, Amazonas, Brazil; 11 Faculdade de Ciências Agrárias (FCA), Universidade Federal do Amazonas, Manaus, Amazonas, Brazil; Instituto Leonidas e Maria Deane / Fundacao Oswaldo Cruz, BRAZIL

## Abstract

The fungi present in the breeding waters of mosquitoes have been scarcely investigated. This work explored the diversity of cultivable fungi present in the breeding sites of the South American malaria vector mosquito *Anopheles darlingi*. Water samples were collected from four sites located in the municipalities of Coari and São Gabriel da Cachoeira and four different culture media were used for the isolation of fungi. Two-hundred-and-six fungal strains were isolated and morphologically similar fungi were grouped into 30 morphotypes. Their taxonomic identities were assigned by macro and microscopic observations and sequencing of rDNA internal transcribed spacers (*ITS1-5*.*8S-ITS2*). Representatives of 26 morphotypes were identified at the genus level, one only at the family level, and three were not identified. The identified morphotypes belong to the phyla, Ascomycota (80.6%), Basidiomycota (11.7%), and Mucoromycota (2.4%), distributed in five classes, ten orders, 25 families, and 26 genera. This study fills a considerable knowledge gap about the fungi present in the breeding sites of *An*. *darlingi* mosquitoes.

## Introduction

The Amazon basin has the largest volume of fresh water on the planet [1]. In that tropical environment biodiversity abounds, and much remains to be explored about species diversity and the ecological relationships among them. The mosquito *Anopheles darlingi* Root, 1926, is the main malaria vector in the Amazon region [2–4]. Despite the importance of malaria in the Amazon and the tropics, little effort has been made to study and identify the fungi associated with these vector mosquitoes [5, 6], especially compared to studies of the bacteria associated with them [7–11].

Although the complex mosquito-associated microbiota is made up of bacteria, fungi, protists, viruses, and nematodes, fungi have been largely neglected [12]. Fungi are an important part of the mosquito larval diet, providing long-chain polyunsaturated fatty acids and phytosterols [13]. Furthermore, fungi synthesize and secrete volatile molecules that attract gravid female mosquitoes and signal suitable oviposition sites [14, 15]. Concerning public health applications, entomopathogenic fungi as well as fungi-derived enzymes and toxins have been used effectively in mosquito control [16, 17], providing alternatives to conventional chemically-synthesized insecticides [16, 18, 19]. Fungi with low levels of pathogenicity can modulate the immune system of mosquitoes, interfering with the development of malaria parasites and other pathogens [20].

The fungi present in the aquatic habitats of mosquito larvae may provide the appropriate means to develop and implement biological control measures against malaria vectors [21–23], however, few mosquito-fungi interactions have been characterized. Therefore, this study aimed to explore the diversity of cultivable fungi present in the aquatic habitats of *Anopheles darlingi* larvae in two malaria-endemic municipalities in the state of Amazonas, and to fill a considerable gap in knowledge of the fungi present in the breeding sites of this important malaria vector.

## Methods

### Sampling sites and collection

Water samples were collected from permanent *An*. *darlingi* breeding sites identified by the Epidemiological Surveillance of the Municipal Health Secretariat from the municipalities of Coari and São Gabriel da Cachoeira, in the State of Amazonas—Brazil (Table 1), with official authorization (21263–1) granted by the Biodiversity Authorization and Information System (SISBIO) of the Brazilian Ministry of the Environment (MMA). Two sites were selected in each municipality and from each site water samples were collected at four equidistant points (5 m from each other) (S1 Fig). Water samples were collected on the surface of the breeding sites in 1-liter sterile glass bottles and kept at 4°C during transportation to the Laboratory of Bioassays and Microorganisms of the Amazon (LaBMicrA) of the Federal University of Amazonas (Universidade Federal do Amazonas, UFAM).

**Table 1:** Location and characteristics of the sites for collecting water samples from *Anopheles darlingi* breeding sites.

Site	Location	Characteristics	GPS Coordinates		Date
			Latitude (S)	Longitude (W)	(Month/year)
	**Coari**				
1 -	Sítio do Gordo (C1)	- Permanent dam area, with fish and vegetation on the margins	4°06’43.7"	63°07’43.6"	02/2017
2 -	Sítio João do Boi (C2)	- Permanent natural lake, with fish and vegetation on the margins.	4°06’56.6"	63°08’34.4"	02/2017
	**São Gabriel da Cachoeira**				
3 -	Sítio Matador (S1)	- Permanent fishpond with fish and no vegetation on the margins.	0°6’54.873’’	67°5’12.859’’	02/2017
4 -	Sítio do Pelado (S2)	- Permanent natural lake, with vegetation on the margins and no fish.	0°7’6.866’’	67°4’24.576’’	02/2017

### Isolation of fungi

For the isolation of filamentous fungi, 100 μl aliquots of sample materials were transferred to Petri dishes (90x15 mm) containing one of the following culture media: AVA (10 g/l oats, 15 g/l agar, 4 g/l dextrose, 4 g/l yeast extract, and 10 g/l malt extract), PDA + L (200 g/l potato, 20 g/l dextrose, and 15 g/l of agar plus 2 g/l yeast extract) [24], ISP2 (10 g/l agar, 10 g/l starch, 4 g/l dextrose, 4 g/l yeast extract, and 10 g/l extract malt), or SDAY (15 g/l agar, 40 g/l dextrose, 10 g/l yeast extract, and 10 g/l peptone). Inoculation in each medium was in triplicate, and all media were supplemented with tetracycline and ampicillin (50 μg/ml each) to inhibit bacterial growth.

The plates were incubated at 26°C for up to 20 days and monitored daily. Beginning on the fifth day of incubation, visible fungal colonies were transferred individually to new plates with the same culture medium. Successive reinoculations were performed until pure cultures were obtained. All purified cultures were preserved at -80°C in 20% glycerol. Those with conidia or spores were also preserved in distilled water [25]. The isolated and preserved strains were deposited in the LaBMicrA/UFAM work collection and registered under the SisGen (National System for the Management of Genetic Heritage and Traditional Knowledge Associated) number AD64E07.

### Morphological analysis

Morphological identifications followed taxonomic keys [26–31], according to the macro and micromorphological characteristics observed. Macroscopic characters included, color, shape, colony diameter, texture, mycelium elevation, and pigment diffusion.

For microscopic examinations, strains were inoculated in a Petri dish at three equidistant points, 1 cm from the edge. Coverslips were placed on top of two of these inocula, leaving the third as a visual control of the colonies. Whenever differentiation from the vegetative mycelium was observed, one coverslip was removed and stained with lactophenol blue to confirm the appearance of the reproductive structures. Additional incubation time was allowed before removing the second micro-cultivation coverslip and staining, when necessary. The vegetative and reproductive microstructures were examined and microphotographed using the Axio Lab. A1 trinocular microscope (Zeiss) with 400X and 1000X magnification. Fungal strains that exhibited similar morphological characteristics were grouped into morphotypes. At least 5% of the strains of each morphotype were randomly chosen to perform rDNA sequencing.

### DNA extraction, rDNA amplification, and sequencing

Each fungal strain was grown in 125 ml Erlenmeyer flasks containing 50 ml of Potato Dextrose Broth (PDB) medium for 24–72 h at 26°C and 120 rpm. The mycelium was separated by vacuum filtration on Whatman paper, No. 4, and crushed with SilicaFlash Irregular Silica Gel G60 (SiliCycle) to lyse the cells. Genomic DNA was extracted with a ZR Fungal/Bacterial DNA MiniPrep kit (Zymo Research, USA), according to the manufacturer’s instructions. The DNA quantity and quality were assessed by optical density measurements (NanoDrop 2000, Thermo Scientific, USA), and gel electrophoresis, respectively.

Approximately 700 bp DNA fragments including internal transcribed spacers (*ITS1-5*.*8S-ITS2*) of the *rDNA* were amplified using primers ITS1 and ITS4 [32]. The amplification reaction had the final volume of 25 μl containing: 0.5 μl of each primer at 10 pmol (Invitrogen), 1 μl DNA at 50–100 ng/μl, 2.5 μl 10x EasyTaq Buffer with Mg_2_ (TransGen Biotech Co.), 0.3 μl EasyTaq DNA Polymerase at 5 U/μl (TransGen Biotech Co.), 1 μl dNTP at 2.5 mM (TransGen Biotech Co.), and 19.2 μl of milli-Q water. PCR was performed using a BioRad S1000 thermal cycler (BioRad Laboratory, CA) with an initial incubation at 94°C for 3 min, followed by 30 cycles of [94°C for 30 s, 58°C for 30 s, and 72°C for 1 min], and a final incubation at 72°C for 5 min.

Amplicons were visualized by electrophoresis on 1.5% agarose gel stained with GelRed (Invitrogen). PCR products were treated with ExoSAP Ilustra—ExoProStar (GE Healthcare) prior to sequencing reactions using BigDye Terminator v.3.1 Cycle Sequencing Kit (Applied Biosystems) and a 3500 Genetic Analyzer (Applied Biosystems) sequencer. Sequencing reactions were performed using primers ITS1 and ITS4 [31].

### Sequence analysis and taxonomy assignment

Consensus sequences were assembled using DNA Sequence Assembly BASER Software v.4.5.0 (http://www.dnabaser.com/index.html) and all sequences generated in this study were deposited in the NCBI GenBank database (accession numbers MZ781245—MZ781299). The sequences were then compared with the sequences stored in GenBank at the NCBI (National Centre for Biotechnology Information) using the BLASTn algorithm (https://blast.ncbi.nlm.nih.gov/Blast.cgi).

Alignments were performed using the MAFFT online interface [33], followed by manual adjustments using MEGA v.7 [34]. Maximum likelihood analyses were performed using RAxMLHPC2 v.8.2.8 [35] in XSEDE. Phylogenetic trees were projected in FigTree 1.4 [36].

### Data statistical analysis

For statistical analysis, we used raw data detailing isolates from each breeding site and culture medium. We computed Shannon-Wiener (H’) diversity and Jaccard similarity indices to characterize species richness and community across breeding sites, with dendrograms illustrating point clustering to visualize these relationships. The normality of the data was assessed using the Shapiro-Wilk test. Due to frequent deviations from the normal distribution, we used the non-parametric Kruskal Wallis test to assess differences in fungal richness between sites, followed by Dunn post-hoc test for pairwise comparisons, with a significance level of 95% (p ≤ 0.05). Non-metric multidimensional scaling (nMDS) was performed using Bray-Curtis dissimilarity index to analyze species distribution patterns and similarities among sites. To determine the significance of differences between clusters, we performed a Similarity Analysis (ANOSIM) using R [37]. In addition, a Venn diagram was generated with the tool Interacti Venn developed by Heberle [38] to visualize the overlap and unique components of fungal communities across breeding sites.

## Results

### Richness, diversity, and characterization of cultivable fungi from *Anopheles darlingi* breeding sites

A total of 206 fungal strains have grown from *An*. *darlingi* larvae breeding waters, collected in Coari (sites C1 and C2) and São Gabriel da Cachoeira (sites S1 and S2). The culture media AVA (oats, agar, dextrose, yeast extract, and malt extract), PDA + L (potato, dextrose, and agar plus yeast extract), ISP2 (agar, starch, dextrose, yeast extract, and extract malt), and SDAY (agar, dextrose, yeast extract, and peptone) used in this work supported the growth of many fungi. Small differences were observed when comparing the number of isolates successfully grown on each medium, SDAY (n = 57), PDA+L (n = 52), ISP2 (n = 50), and AVA (n = 47)—(S1 Table), indicating their applicability in the recovery of fungi from aquatic freshwater habitats. The C1 site (n = 107) yielded the highest number of isolates, followed by the sites S2 (n = 45), C2 (n = 44), and S1 (n = 10) (Table 2).

**Table 2:** Identification of taxa and allocation at the collection sites of fungi isolated from the waters of *Anopheles darlingi* larvae breeding sites in the Brazilian Amazon.

Class	Taxon	No of isolated strains	Distribution of isolates at collection sites			
			C1	C2	S1	S2
Agaricomycetes	*Emmia* ^*1*^	4	0	0	0	4
	*Hypomontagnella* ^*1*^	7	3	0	0	4
	*Peniophora* ^*1*^	5	5	0	0	0
	*Trametes* ^*1*^	8	3	2	1	2
Dothideomycetes	*Cladosporium* ^*2*^	2	2	0	0	0
	Cucurbitariaceae ^*1*^	2	1	1	0	0
	*Epicoccum* ^*1*^	1	1	0	0	0
	*Hongkongmyces* ^*1*^	5	2	3	0	0
	*Microsphaeropsis* ^*1*^	19	16	3	0	0
	*Nigrograna* ^*1*^	1	1	0	0	0
	*Ochronis* ^*2*^	12	8	3	0	1
	*Paraconiothyrium* ^*8*^	23	19	0	3	1
	*Pyrenochaetopsis* ^*2*^	4	4	0	0	0
Eurotiomycetes	*Aspergillus* ^*5*^	12	5	2	2	3
	*Penicillium* ^*2*^	14	2	1	1	10
	*Talaromyces* ^*4*^	8	2	4	1	1
Mucoromycetes	*Gongronella* ^*1*^	5	1	1	0	3
Sordariomycetes	*Albifimbria* ^*1*^	1	1	0	0	0
	*Chrysoporthe* ^*1*^	4	2	1	0	1
	*Cytospora* ^*3*^	3	0	3	0	0
	*Diaporthe* ^*3*^	6	1	2	0	3
	*Eutypella* ^*2*^	2	0	2	0	0
	*Fusarium* ^*4*^	19	12	4	0	3
	*Hyphodermella* ^*1*^	3	1	1	0	1
	*Sarocladium* ^*2*^	5	1	1	0	3
	*Striaticonidium* ^*1*^	3	1	1	0	1
	*Trichoderma* ^*2*^	17	6	6	1	4
	NID1 ^1^	6	6	0	0	0
	NID2 ^2^	1	0	1	0	0
	NID3 ^3^	4	1	2	1	0
Total	30	206	107	44	10	45

NID (unidentified)—refers to fungi grouped into morphotypes that could not be identified by morphological characters or rDNA sequencing. ^n^—Number of specimens sequenced.

Macro and micromorphological characterization of the isolated strains allowed their classification into 30 morphotypes. The diversity of morphotypes grown in different culture media was different. PDA+L supported the highest diversity of morphotypes (n = 23), followed by SDAY (n = 21), AVA (n = 18), and ISP2 (n = 15). Morphological data, together with ribosomal RNA sequences, and analysis of phylogenetic trees identified 26 of the morphotypes at their genus level and one at the family level. In all cases, the molecular data confirmed the morphology-based taxonomy, therefore, for diversity and richness estimation, all strains within a given morphotype were assigned to the same taxon (S2 Table). The ideal outcome would be to assign species-level taxonomy to each queried sequence, however, the limited resolution of the sequenced locus prevented accurate classification at the species level. Three morphotypes proved difficult to cultivate under the conditions described above and their DNA was not sequenced (Table 2). Overall, one in every 6.9 isolated fungal strains was successfully classified at the genus level (S3 Table; S2 Fig).

**Fig 1:**
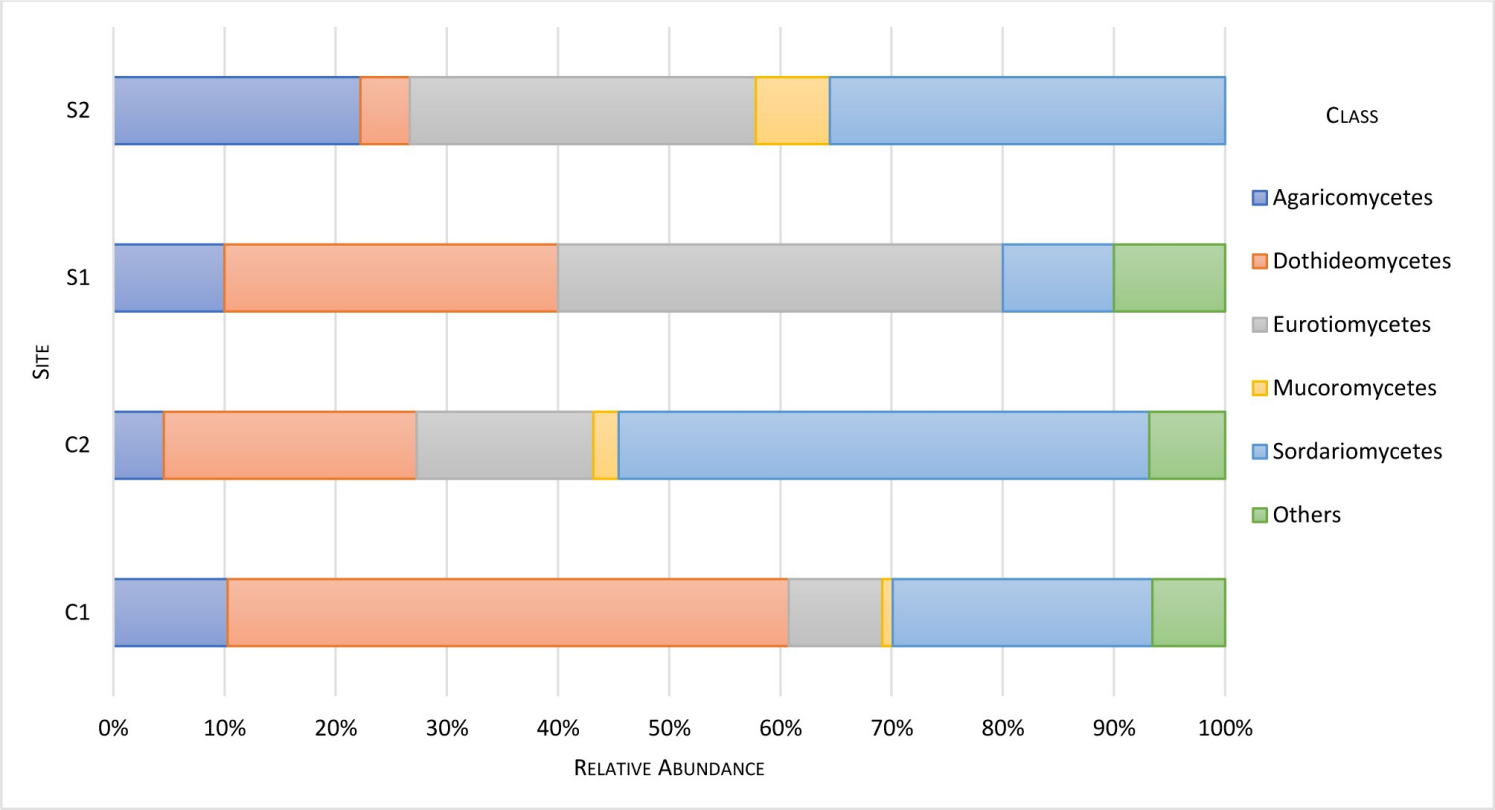
Fungal community composition in different *Anopheles darlingi* breeding sites at class level. Others—refers to fungi that could not be classified at class level using the analyses carried out.

**Fig 2:**
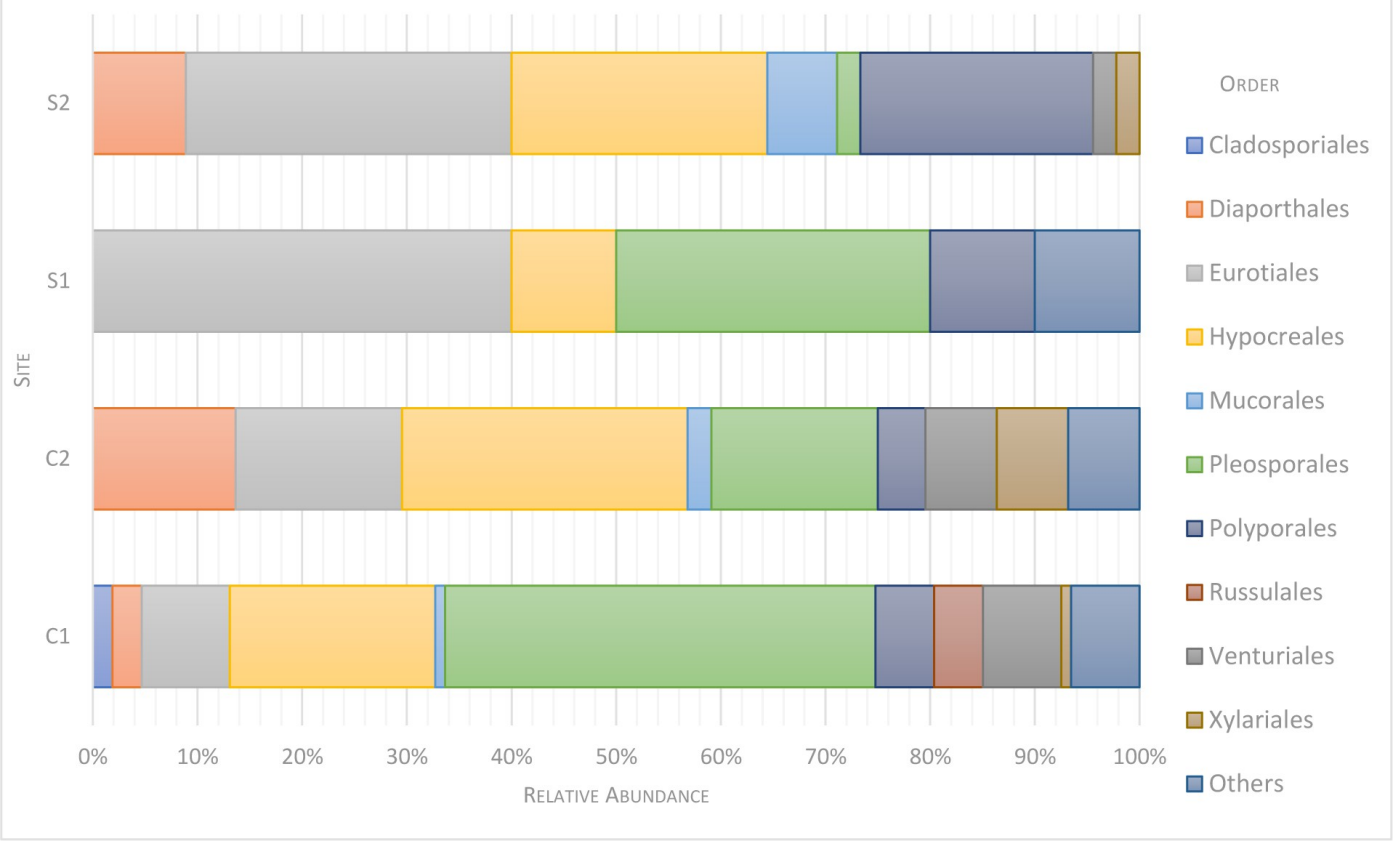
Fungal community composition in different *Anopheles darlingi* breeding sites at the order level. Others—refers to fungi that could not be classified at order level using the analyses carried out.

**Fig 3:**
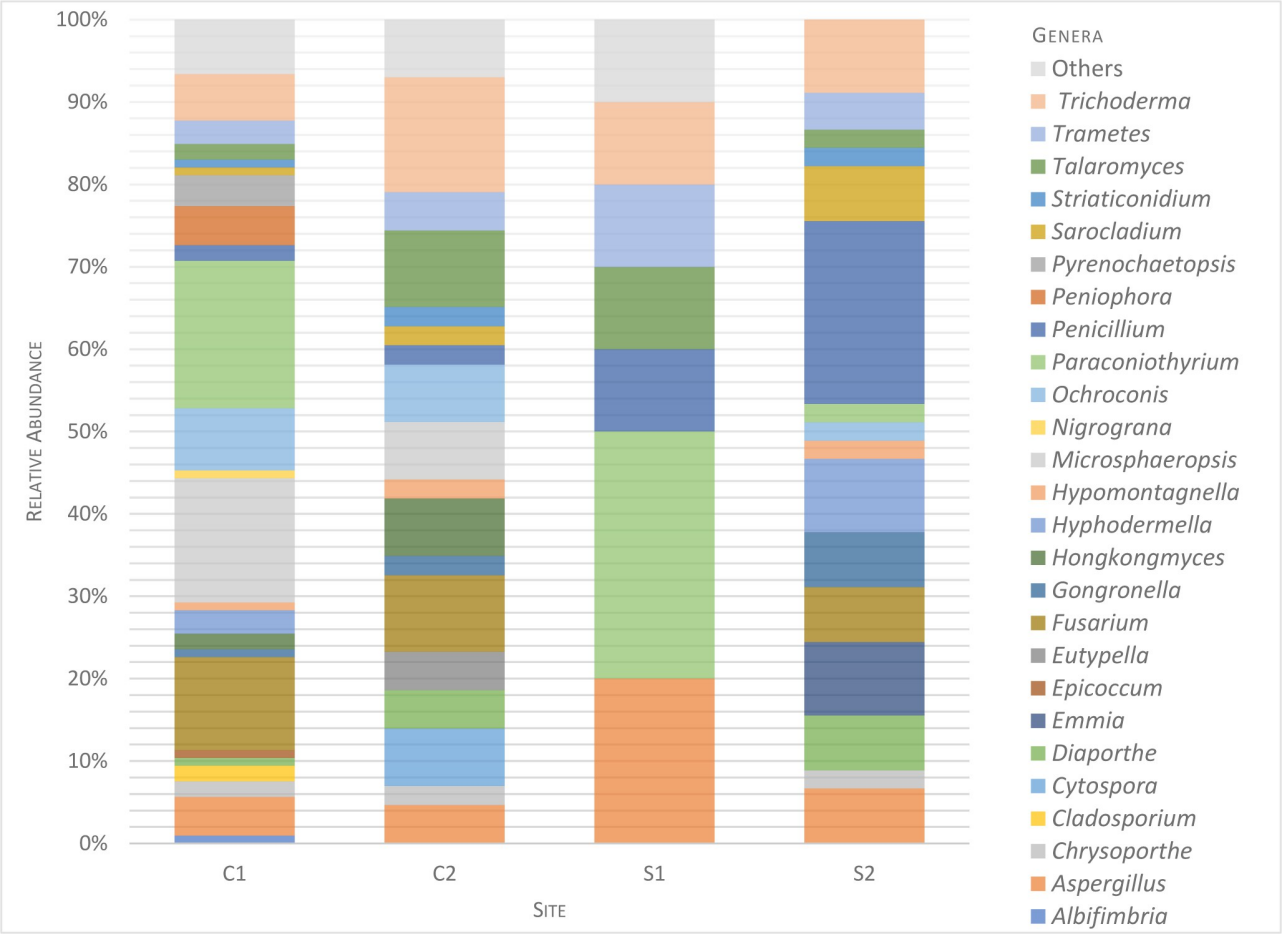
Composition of fungal communities at the genera level at different collection sites. Others—refers to fungi that could not be classified at genera level using the analyses carried out.

The sequenced morphotypes belong to three phyla: 80.6% Ascomycota, followed by Basidiomycota and Mucoromycota, 11.7% and 2.4%, respectively. The three most represented classes were Dothideomycetes, Sordariomycetes, and Eurotiomycetes corresponding to 33.5%, 30.6%, and 16.5%, respectively (Fig 1). Ten orders were identified, the three main ones being: Pleosporales 26.7%, Hypocreales 21.9%, and Eurotiales 16.5% (Fig 2). The sequenced morphotypes had representatives of 25 families and 26 genera (S1 Table). The genus *Paraconiothyrium* was the most prevalent, represented by 11.2% of all isolated fungi strains, followed by *Fusarium* and *Microsphaeropsis*, both with 9.2% (Fig 3).

**Fig 4:**
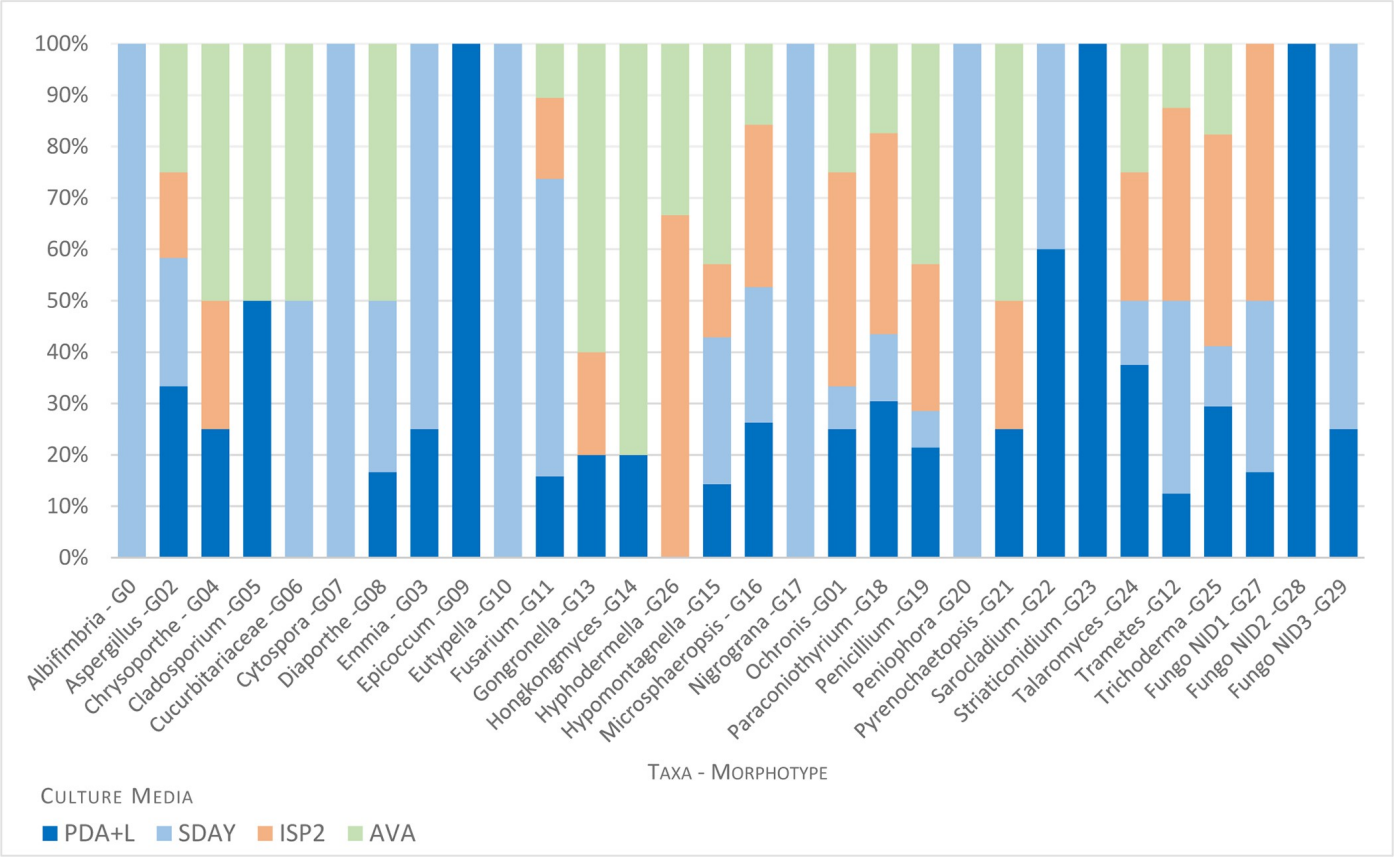
Fungi isolated from *An*. *darlingi* larvae breeding sites in the different culture media AVA, ISP2, PDA + L, and SDAY. Taxonomic assignments of 30 morphotypes (G0-G29) was based on morphological and molecular characterization. Fungo NID (unidentified)1, Fungo NID2 e Fungo NID3—refer to morphotypes that could not be identified by morphology or rDNA sequencing.

Fungi of genera *Albifimbria*, *Cytospora*, *Eutypella*, *Nigrograna*, and *Peniophora* only grew on the SDAY medium, while fungi of genera *Epicoccum*, *Striaticonidium*, and Fungo NID2 grew only on PDA+L medium (Fig 4).

### Fungi taxonomic diversity analysis

The diversity and richness of the fungi, represented by the Shannon (H’) and Chao1 indices, indicated that the samples from the C1 site showed the highest values (Chao1 = 33.5 and H’ = 2.772). Regarding the dominance (Simpson D) and equitability (Equitability_J) indices, the samples from site C2 showed the highest values (D = 0.931 and J = 0.944). In contrast, the S1 site showed the lowest values of diversity (H ’ = 1.834), richness (Chao1 = 12), and dominance (D = 0.82) (S4 Table).

**Fig 5:**
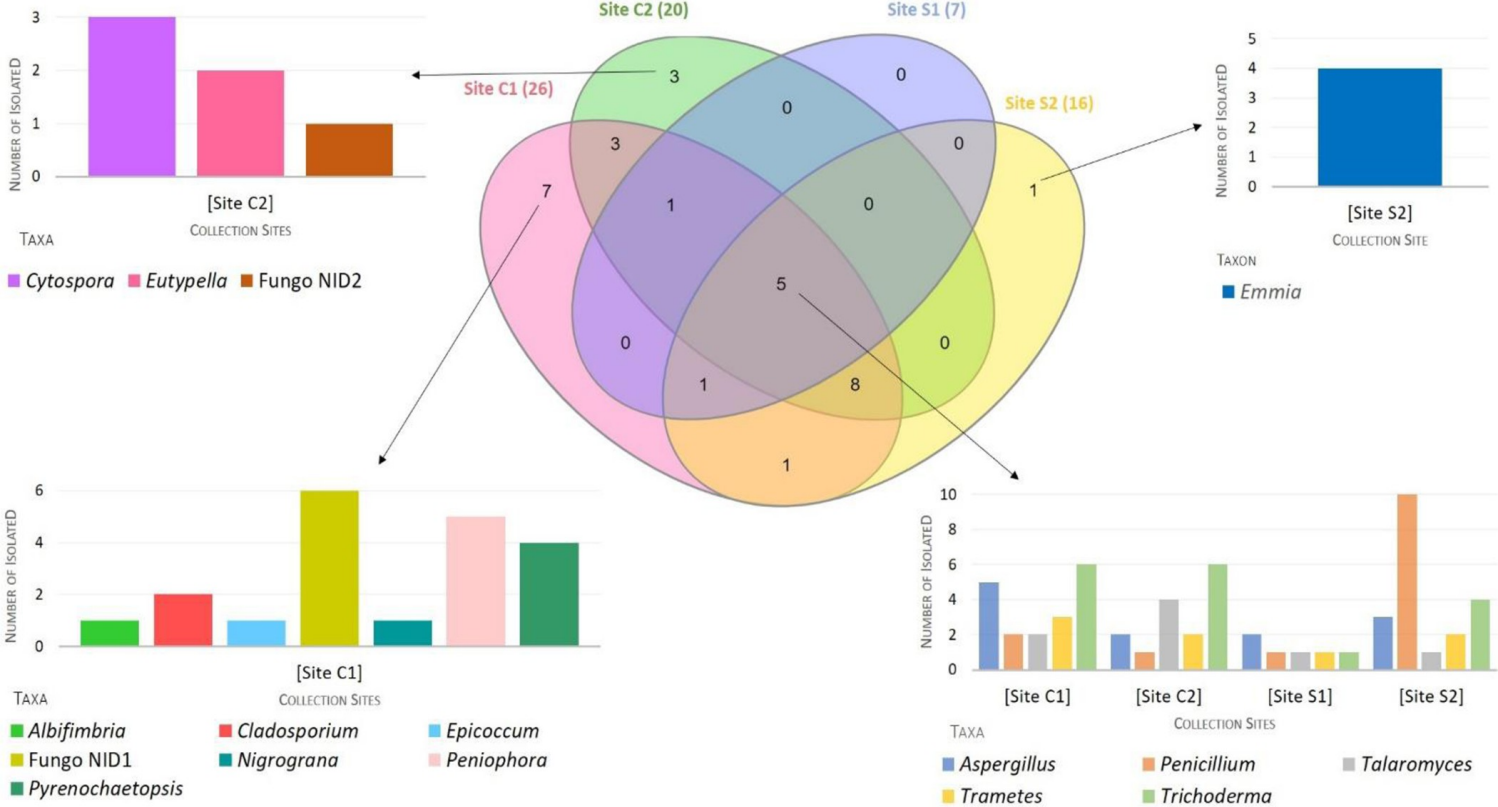
Venn diagram showing the number of fungal taxa shared or exclusive of collection sites C1, C2, S1 and S2. Fungo NID (unidentified)1, Fungo NID2 e Fungo NID3—refer to morphotypes that C1, C2, S1 could not be identified by morphology or rDNA sequencing.

Representatives of five genera (*Aspergillus*, *Penicillium*, *Talaromyces*, *Trametes*, and *Trichoderma*) were shared among the four sampling sites. Seven taxa (*Albifimbria*, *Cladosporium*, *Epicoccum*, *Nigrograna*, *Peniophora*, and *Pyrenochaetopsis* genera, and NID1), were isolated only from site C1. Taxa exclusively isolated from the sites C2 (*Cytospora* and *Eutypella* genera, and ND2) and S2 (*Emmia*) were also observed. All taxa isolated from S1 were also present in at least one other site (Fig 5).

**Fig 6:**
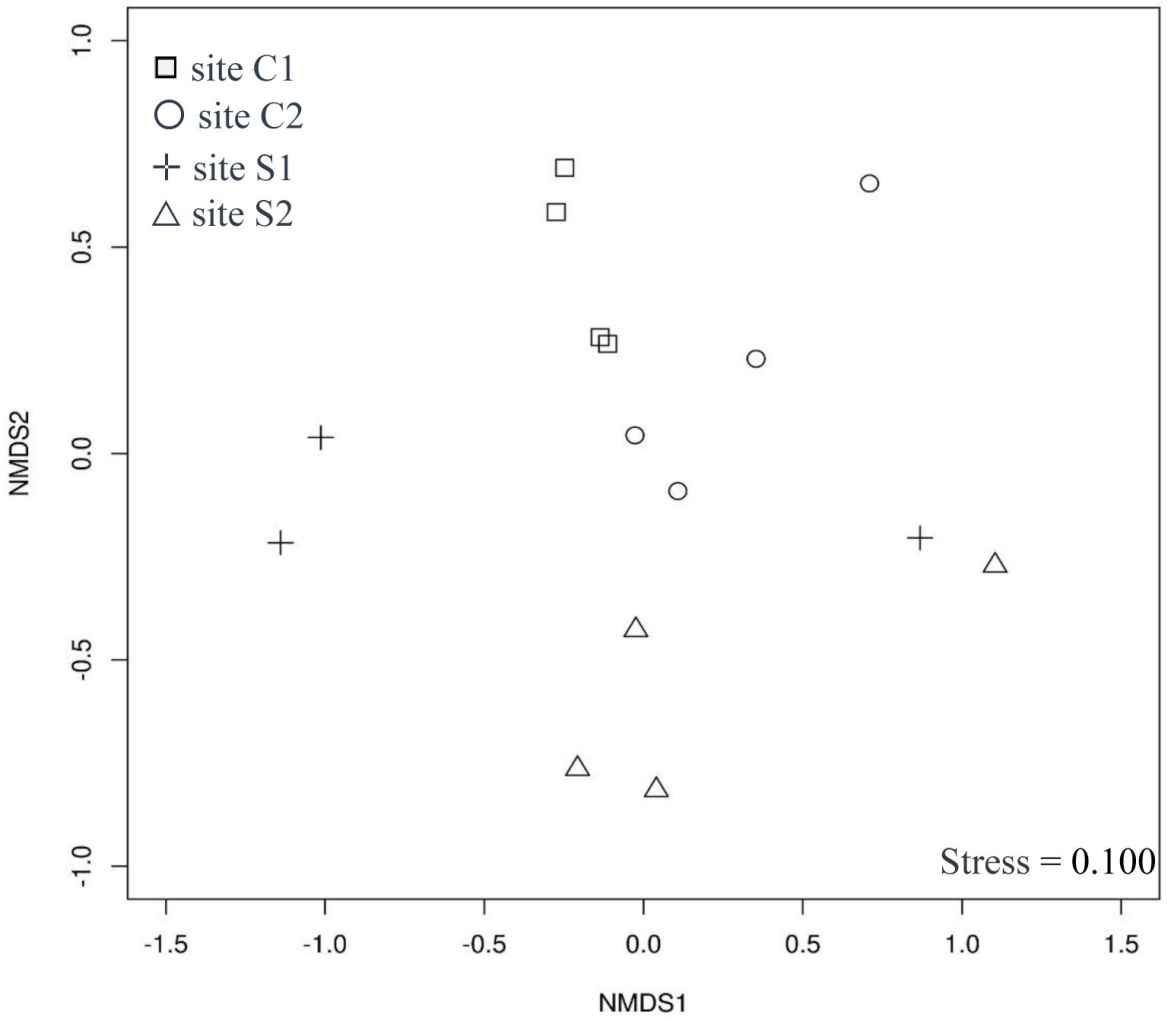
Non-metric multidimensional scaling (nMDS) using the Bray-Curtis distance to show the similarities between the collection sites in relation to the distribution of fungal isolates. In each site, four sub-sites were sampled, however, the S1 site presented isolates in only 3 sub-sites.

The jaccard similarity index identified three groups, with C2 and S2 showing the highest similarity, while S1 showed the greatest divergence (S3 Fig). The Kruskal-Wallis test followed by Dunn’s post hoc comparisons revealed a borderline statistical difference in richness between sites C2 and S2. In contrast to the comparisons between sites C2 and S2, all other pairwise comparisons between sites were significantly different (*p <0*.*05*) (S5 Table). Non-metric multidimensional scaling (nMDS) analysis based on all fungal isolates obtained in the samples and separated by collection site shows that the spatial distribution of the composition of the fungal community varied between the collection sites, with site S1 being less similar among the others. Two sub-sites of the C1 site were the most similar, with the same microbial composition (Fig 6).

## Discussion

*Anopheles* mosquitoes breed in a variety of natural and artificial water bodies, such as riverbanks, streams, lakes, ponds, dams, and fishponds, which generally contain organic matter and aquatic vegetation [39–42]. This aquatic habitat hosts a wide variety of fungi, some of which are associated with mosquitoes throughout their life cycle.

Fungi present in mosquito breeding waters are ingested during larval feeding [13, 43, 44] and attach to their cuticle and external body wall structures. Although most of the microbiota associated with mosquito larvae does not persist after metamorphosis, some fungi are transstadially transmitted across developmental stages, survive metamorphosis, and are inherited by their progeny [20]. Adult mosquitoes can also contact fungi and/or bacteria when standing on or ingesting water from the breeding site immediately after emerging from the pupal stage [40–42]. These adults can introduce or reintroduce fungi into aquatic habitats through contact, urine deposition [45], or during egg laying [46, 47], affecting the microbiota to which larvae are exposed [47].

In this study, we explored and compared the diversity of cultivable fungi in *Anopheles darlingi* breeding water samples from the Brazilian Amazon, collected in the municipalities of Coari (C1 and C2) and São Gabriel da Cachoeira (S1 and S2). Coari is located on the banks of the Solimões River, while São Gabriel da Cachoeira is on the banks of the Negro River, two distinct hydrological basins. Both municipalities are considered highly endemic areas for malaria [48].

The distinct characteristics of the black water of the Negro River, characterized by acidity (pH < 5.0), low productivity, low suspended sediment concentration, and low electrical conductivity, contrast with the white water of the Solimões River, which has a neutral pH (7.0), is rich in nutrients and high in suspended matter and dissolved salts, resulting in a greater diversity of microorganisms [49–52]. According to Fonseca [50] and Tadei [53], the characteristics of black water provide more suitable conditions for the breeding of the malaria vector.

The isolated strains in this study represent only a fraction of all fungi present in the sampled sites. Likely, other fungi could be isolated by collecting and exploring additional samples from the same or other *An*. *darlingi* breeding sites and investigating fungi that grow optimally in other cultivation media. Culture techniques do not capture the full spectrum of microbial diversity; in fact, approximately 99% of naturally occurring microorganisms have been suggested to remain unknown [54–56].

The frequent and heavy rainfall events in the Amazon rain forest, especially during the period when collections were carried out for this study, suggest that some of the fungi found in mosquito breeding waters are transient, carried with plant and soil residues, mainly by rainwater [57, 58]. A longitudinal investigation could reveal details of the fungal population dynamics in the studied breeding sites. Therefore, these results must be interpreted with attentiveness as a number of limitations should be borne in mind.

Ascomycota was highly predominant among the three phyla found in the breeding waters of *An*. *darlingi*, followed by Basidiomycota, while only one morphotype was from the Mucoromycota phylum. This is consistent with previous reports showing that Ascomycota is the largest phylum of fungi, encompassing approximately two-thirds of all described fungal species [59, 60], while Basidiomycota is the second richest phylum in number of species [61]. Both the Ascomycota and Basidiomycota phyla are ubiquitous in nature and are the main phyla found in freshwater environments [60, 62, 63]. The scarcer phylum Mucoromycota consists mainly of mycorrhizal fungi, root endophytes, and plant material decomposers [64]. However, as observed in this work, Mucoromycota is also found in freshwater environments [65].

Similar results were found when analyzing fungi in other mosquito breeding sites in diverse localities worldwide [66, 67]. For example, Tawidian [65], who analyzed the fungal microbiota in samples of *Aedes albopictus* from Manhattan, KS, USA, and water from the larval breeding sites, identified representatives of the phyla Ascomycota (59.5%), Basidiomycota (30.8%), and Mucoromycota (0.46%), among others.

The most represented classes, Dothideomycetes, Sordariomycetes, and Eurotiomycetes, belong to the most abundant Ascomycota phylum. Agaricomycetes and Mucoromycetes were less abundant and were the unique classes found for their respective phyla. Sordariomycetes contain nearly half of all known freshwater Ascomycota, corresponding to approximately 300 out of 620 taxa [68, 69]. The three main classes in this study are also the most abundant in waters from aquatic environments of the High Arctic [70] and other marine environments [57], indicating they are adapted to occupy very different niches.

The C1 site showed the highest richness index, with the highest number of isolates, seven taxa were found exclusively in C1 and 19 were shared with other sites (Fig 5). Such diversity and richness are consistent with the suitable conditions for fungal growth found at the C1 site, a permanent dam with fish and vegetation on the margins and, therefore, rich in organic particles. Water dams such as C1 have characteristics similar to natural environments and are favorable to breeding *Anopheles* [41].

The C2 and S2 sites, the second and third sites in richness, respectively, are permanent natural lakes, with fish and vegetation on the margins, a situation similar to that found in the site C1. The diversity in C2 was 20 taxa, while S2 had 16. The lowest diversity and richness of fungi were attributed to the site S1. This breeding site is a permanent fishpond, without vegetation on the margins and near a deforested area, most affected by anthropic actions, and probably poorer in conditions for fungal survival. In fact, anthropogenic activities affect the biogeochemical properties of breeding sites and, in turn, affect the microbiota of mosquitoes [63, 71, 72]. S1 had only seven taxa: none exclusive, two common to two other sites, and five common to all sites (Fig 5).

Five genera of fungi found in the four sites sampled in this work, *Aspergillus*, *Penicillium*, *Talaromyces*, *Trametes*, and *Trichoderma*, are known to be ubiquitous in the environment [57, 73–75]. Interactions between species of these five genera with mosquitoes have been studied. *Penicillium* species present in the midgut of *Anopheles* made mosquitoes more susceptible to *Plasmodium* infection [76, 77], *Talaromyces* make *Aedes aegypti* more susceptible to dengue virus infection [78], metabolites produced by *Trametes* species showed larvicidal activity against *Ae*. *aegypti* [79], and fungi belonging to the genera *Penicillium*, *Aspergillus*, and *Trichoderma* have larvicidal and adulticidal activities against mosquitoes [19, 80–83].

The present study explored the diversity of cultivable fungi from *An*. *darlingi* breeding sites in the state of Amazonas, Brazil, revealing rich and diverse fungal communities in more natural freshwaters and poorer diversity in the anthropic influenced one. The knowledge generated by this study could be leveraged to achieve a more holistic understanding of mosquito biology and its associated microbiome. Similar to bacterial endosymbionts, mosquito-associated fungi could harbor both beneficial and antagonistic traits. [44, 46, 84–86]. Fungi from the genera found in this work have been investigated as potential agents against *Anopheles* larvae. However, native fungus isolates may offer a superior alternative to the introduction of foreign biocontrol agents, as they can be better adapted to infect and kill local mosquitoes and survive in the Amazonian local environmental conditions, with high temperatures and abundant rainfall [87]. In fact, extracts of Amazonian fungi isolated during this investigation, *Albifimbria* 1160 and *Diaporthe* 1203, are active in the killing of mosquito larvae [76].

## Supporting information

S1 Fig Surface water sampling/collection locations. (DOCX)

S2 Fig Phylogenetic trees of fungi isolated from *An*. *darlingi* breeding sites. (DOCX)

S3 Fig Dendrogram of fungi isolated from *An*. *darlingi* breeding sites. (DOCX)

S1 Table List of all fungi isolated from *An*. *darlingi* breeding sites. (DOCX)

S2 Table Morphotype diversity of isolated fungi. (DOCX)

S3 Table Taxonomic classification of fungi isolated from *An*. *darlingi* breeding sites. (DOCX)

S4 Table Diversity estimates found at different collection sites. (DOCX)

S5 Table Kruskal-Wallis test with Dunn’s post hoc tests to verify the difference in richness among the collection sites. (DOCX)
