# Supplementary material for: Fungi from Anopheles darlingi Root, 1926, larval breeding sites in the Brazilian Amazon
Source: PLoS One. 2024 Dec 5;19(12):e0312624. doi: 10.1371/journal.pone.0312624 (PMC11620424; doi:10.1371/journal.pone.0312624)

**Supplementary Figure 1.** Surface water sampling/collection locations. **Top** - Map showing the sampling sites in Coari and São Gabriel da Cachoeira - Amazonas/Brazil. The map is based on open access files obtained from the Brazilian Institute of Geography and Statistics (Instituto Brasileiro de Geografia e Estatística–IBGE). **Bottom** - Photos of the collection sites: A) Coari 1 - Gordo site (C1), B) Coari 2 - João do Boi site (C2), C) São Gabriel da Cachoeira 1 - Matador site (S1), D) São Gabriel da Cachoeira 2 - Pelado site (S2). All sites are *Anopheles darlingi* larval habitats.


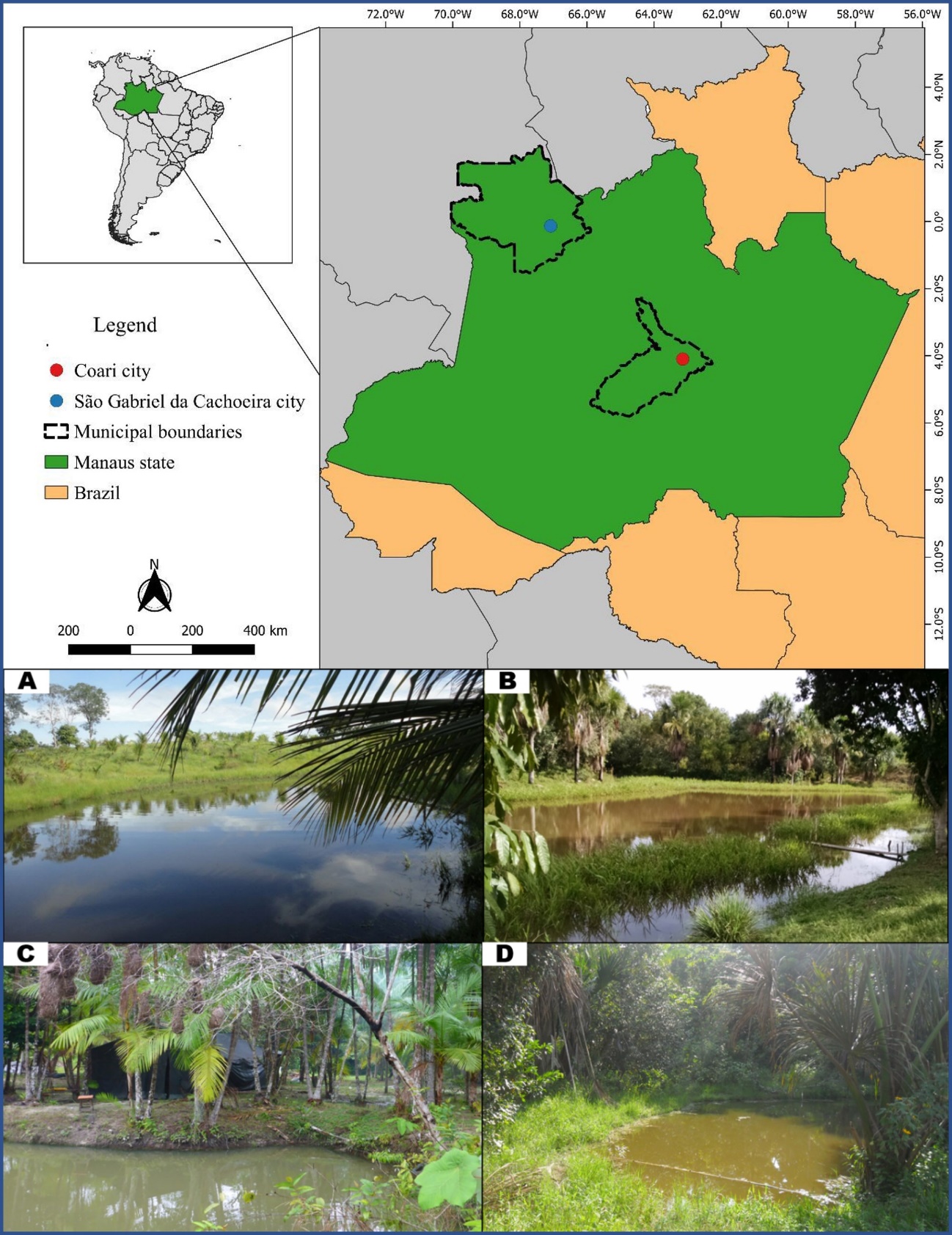

Supplement: S1 Fig — (DOCX) [file pone.0312624.s001.docx]
