# Supplementary material for: Fungi from Anopheles darlingi Root, 1926, larval breeding sites in the Brazilian Amazon
Source: PLoS One. 2024 Dec 5;19(12):e0312624. doi: 10.1371/journal.pone.0312624 (PMC11620424; doi:10.1371/journal.pone.0312624)

**Supplementary Figure 2.** Phylogenetic trees of fungi isolated from *An. darlingi* breeding sites in the municipalities of Coari (C1 and C2) and São Gabriel da Cachoeira (S1 and S2) and their closest relatives.


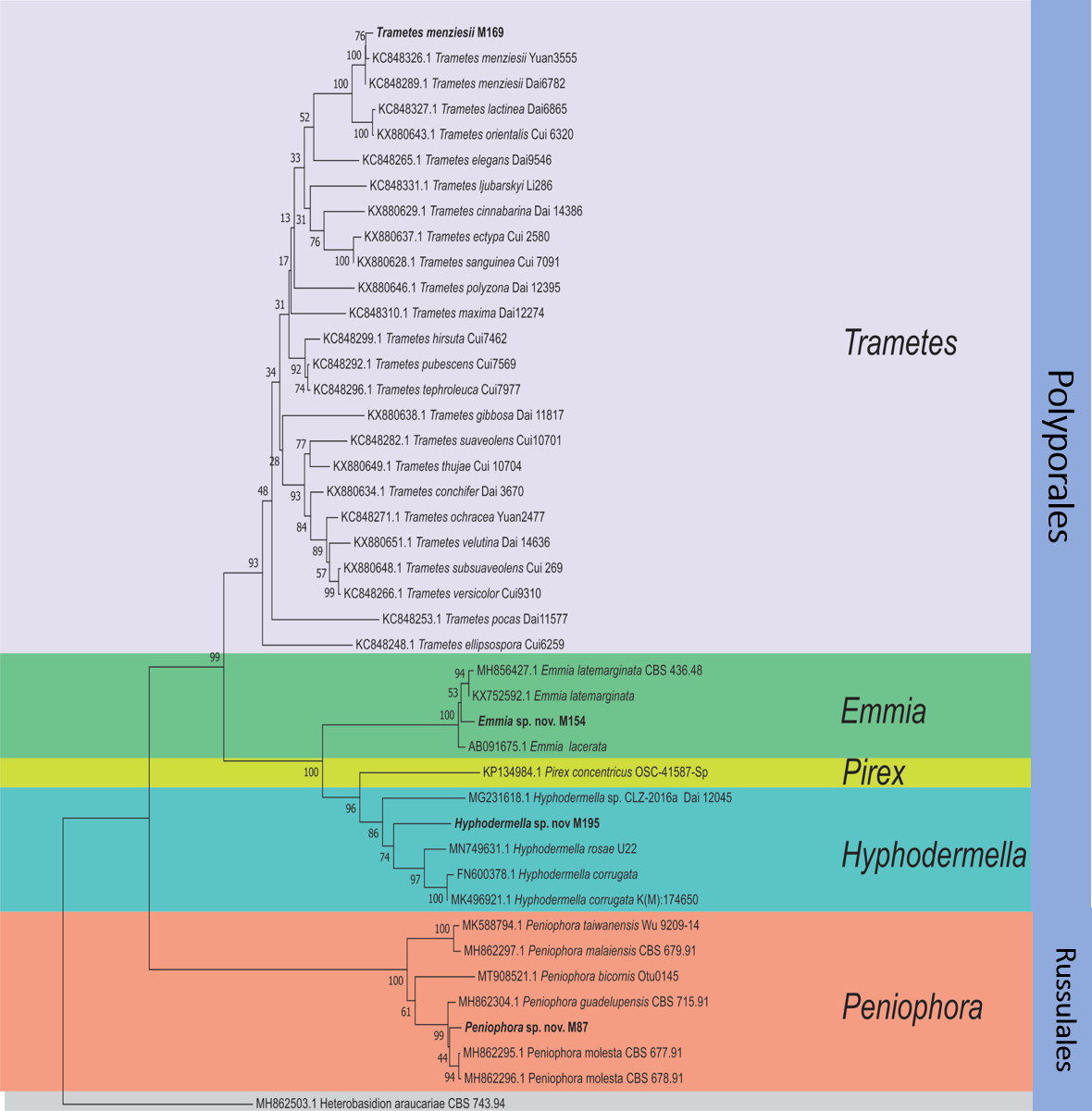


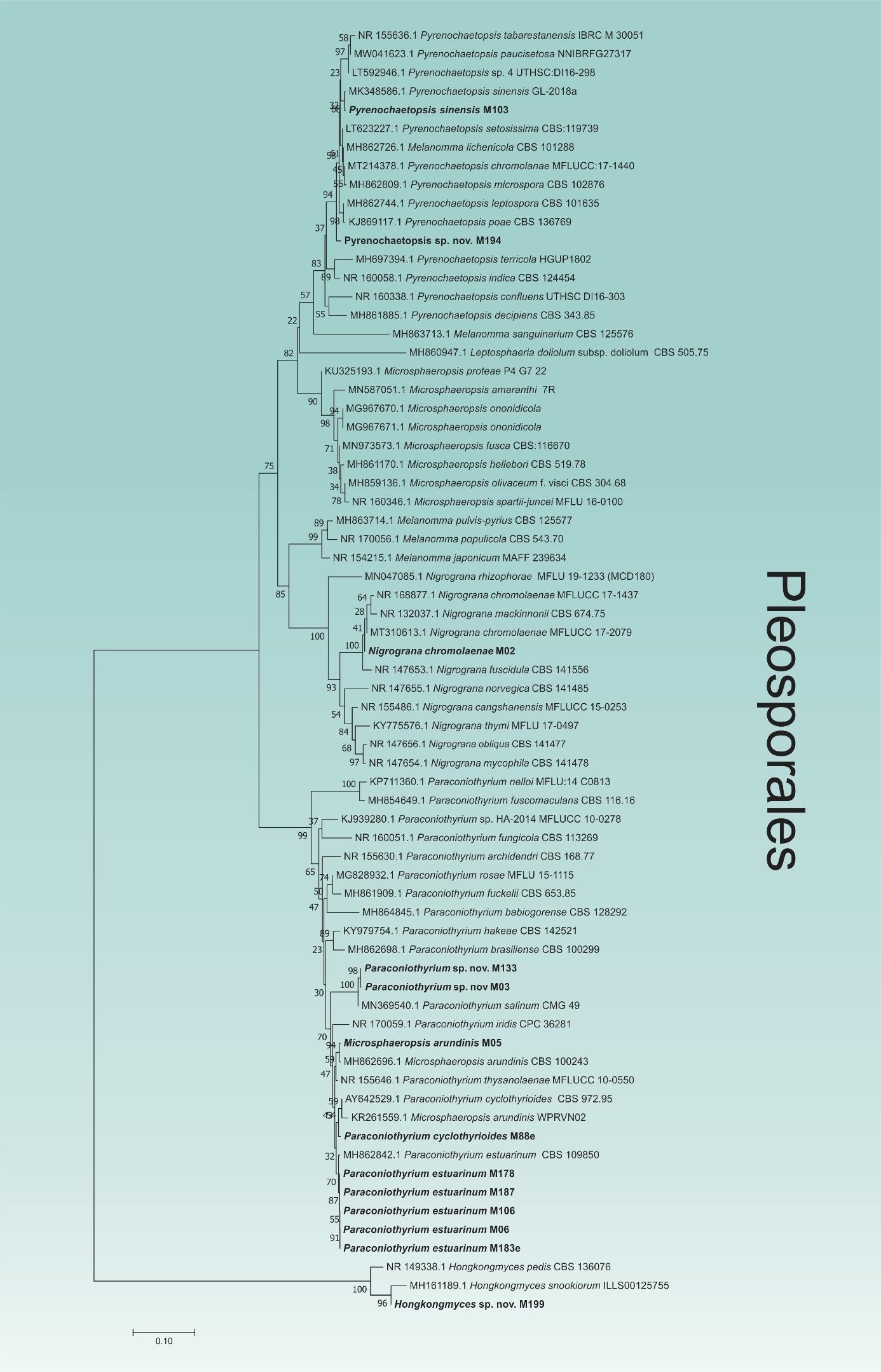


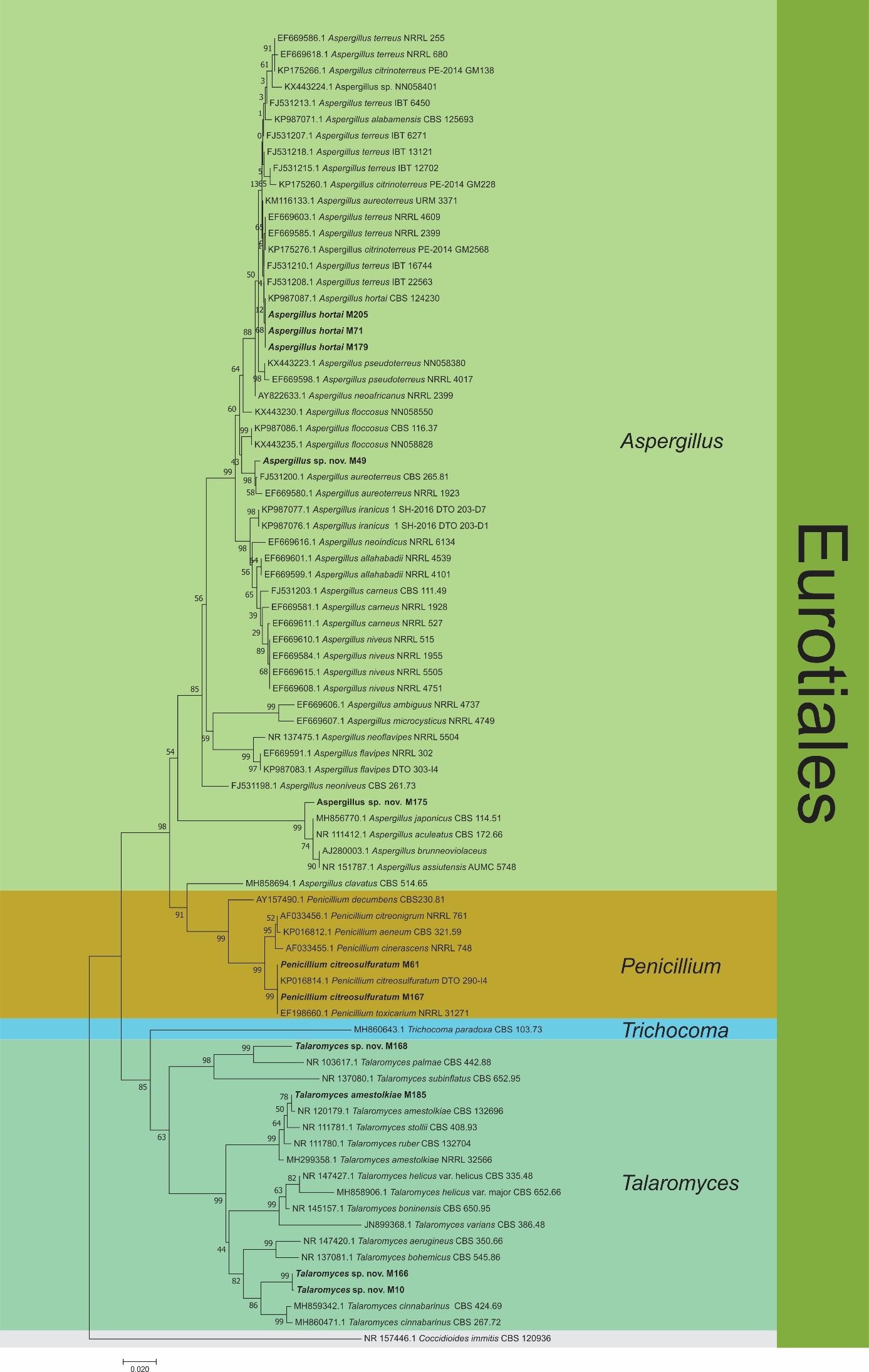


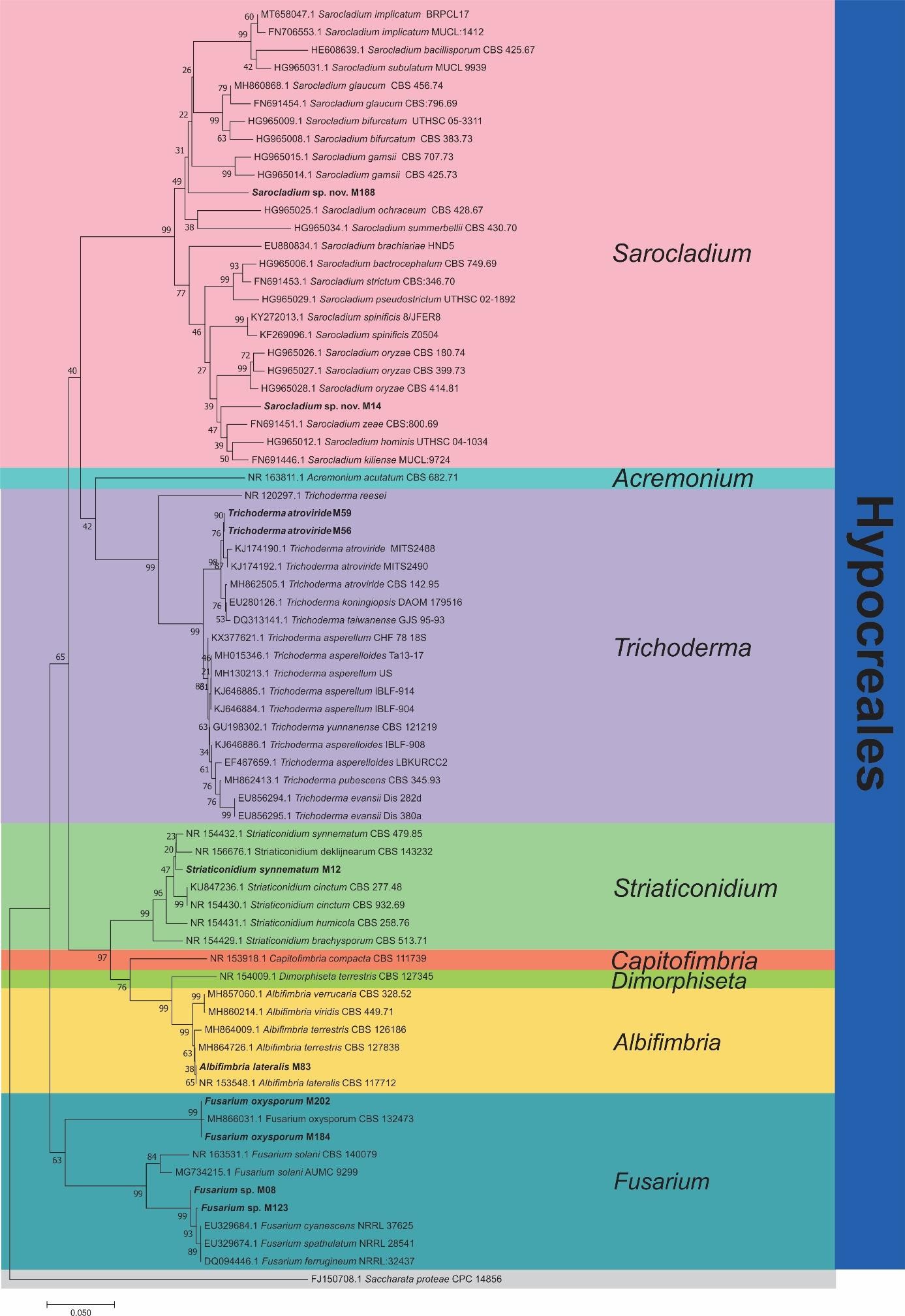


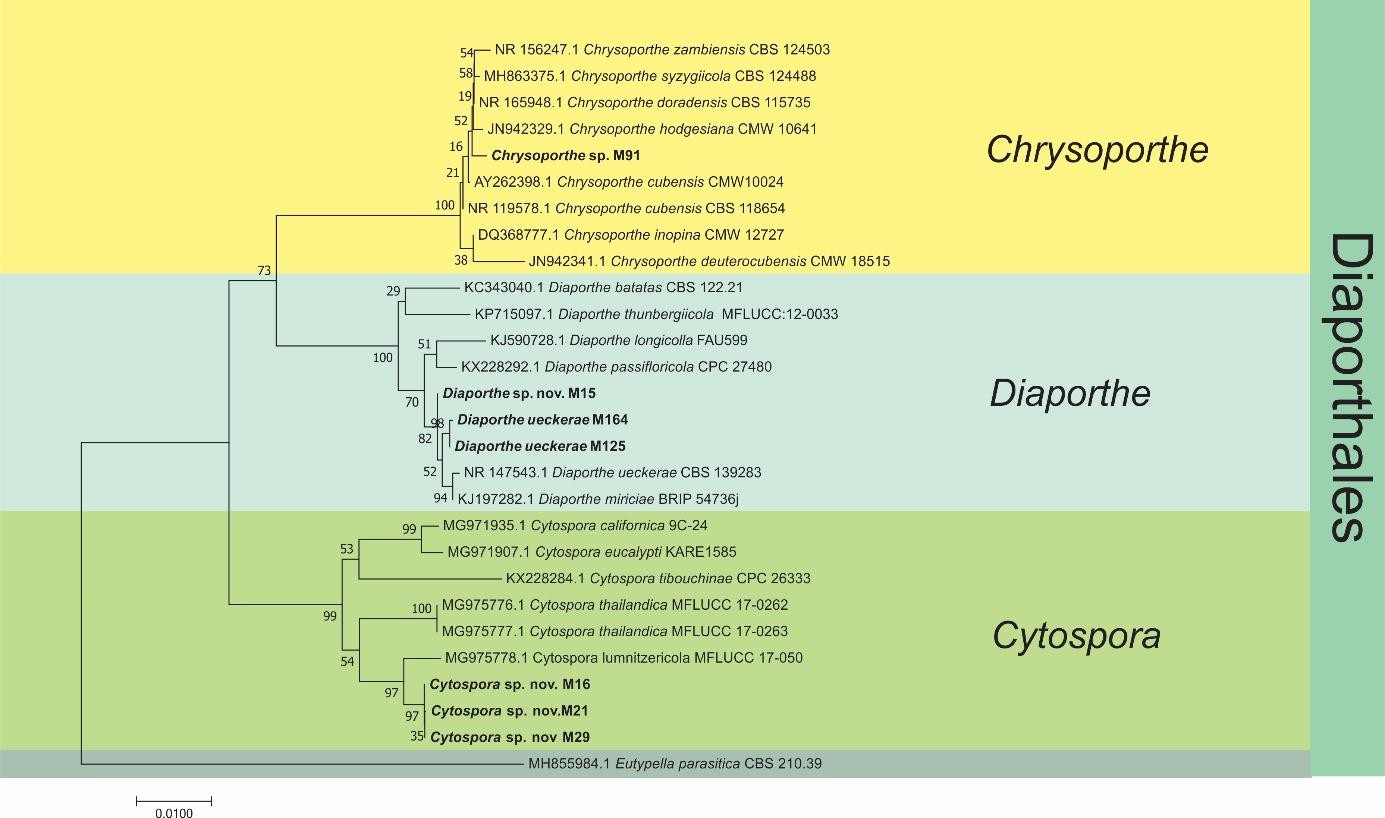

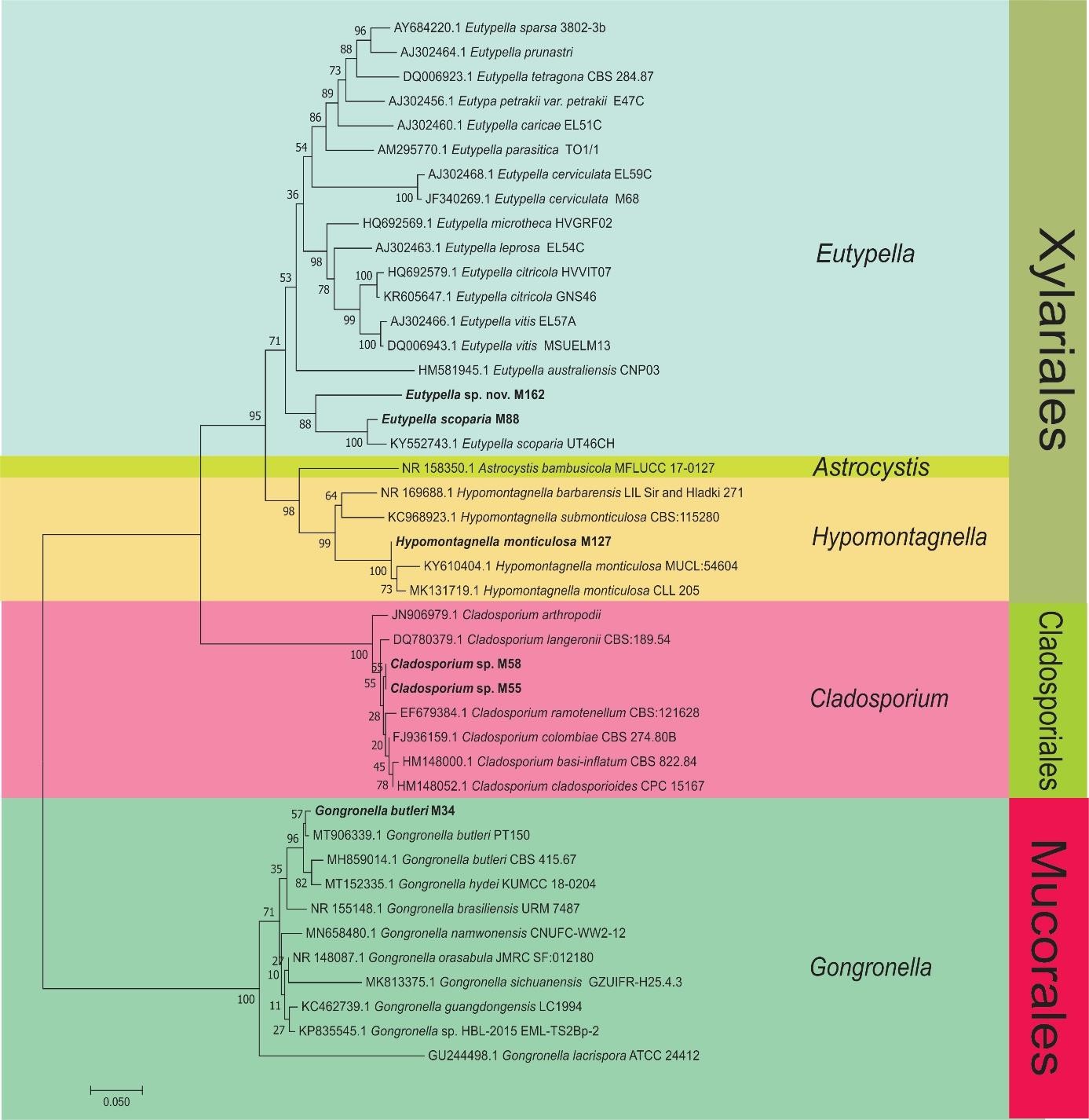


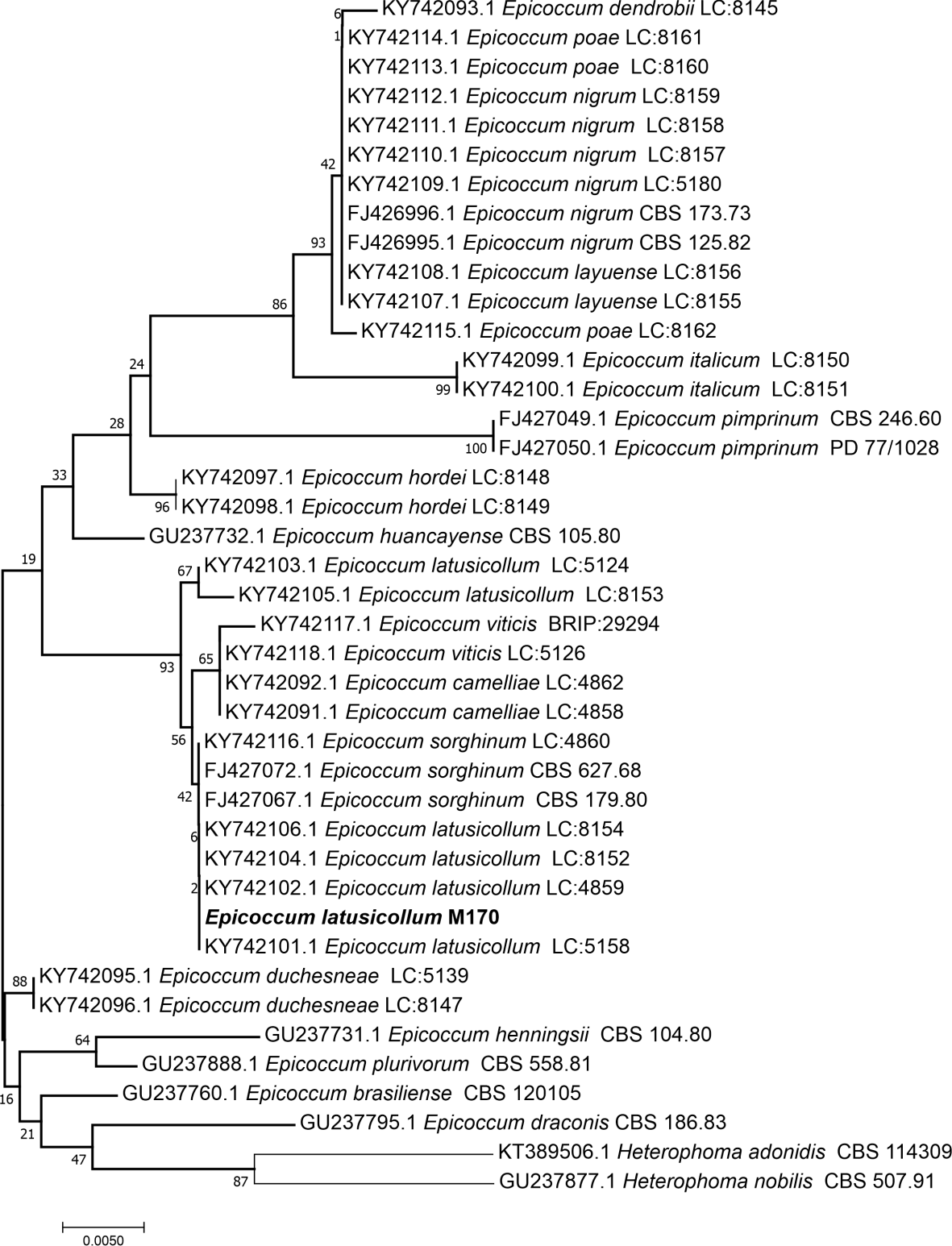


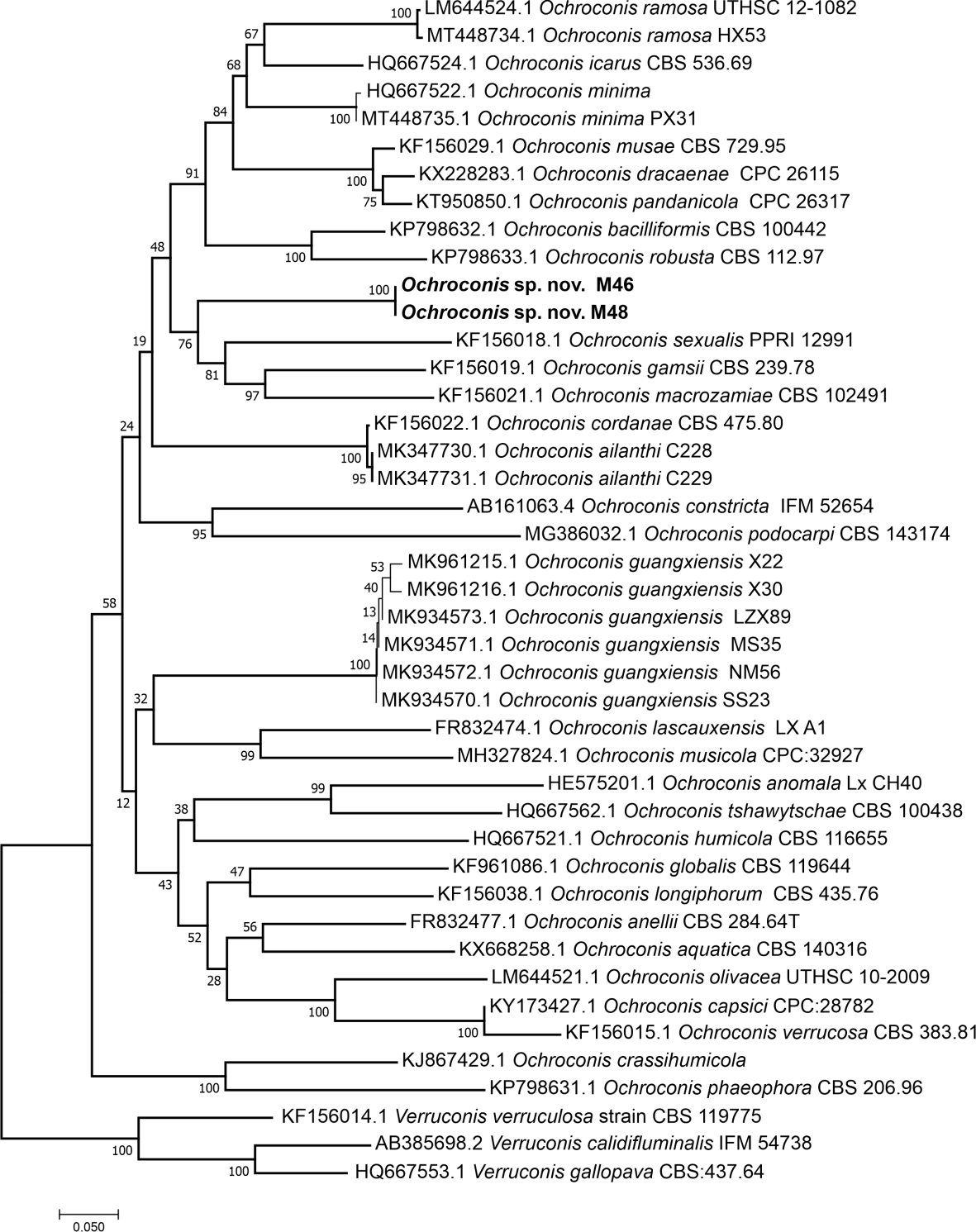

Supplement: S2 Fig — (DOCX) [file pone.0312624.s002.docx]
