# Supplementary material for: Fungi from Anopheles darlingi Root, 1926, larval breeding sites in the Brazilian Amazon
Source: PLoS One. 2024 Dec 5;19(12):e0312624. doi: 10.1371/journal.pone.0312624 (PMC11620424; doi:10.1371/journal.pone.0312624)

**Supplementary Figure 3.** Dendrogram of fungi isolated from the aquatic freshwater habitat of *An. darlingi* at different collection sites C1, C2, S1 and S2, according to the Jaccard similarity. The analysis of the Jaccard similarity index showed the formation of three groups, in which C2 and S2 sites are the most similar to each other, while the S1 is the most different site, being more distant from the others.


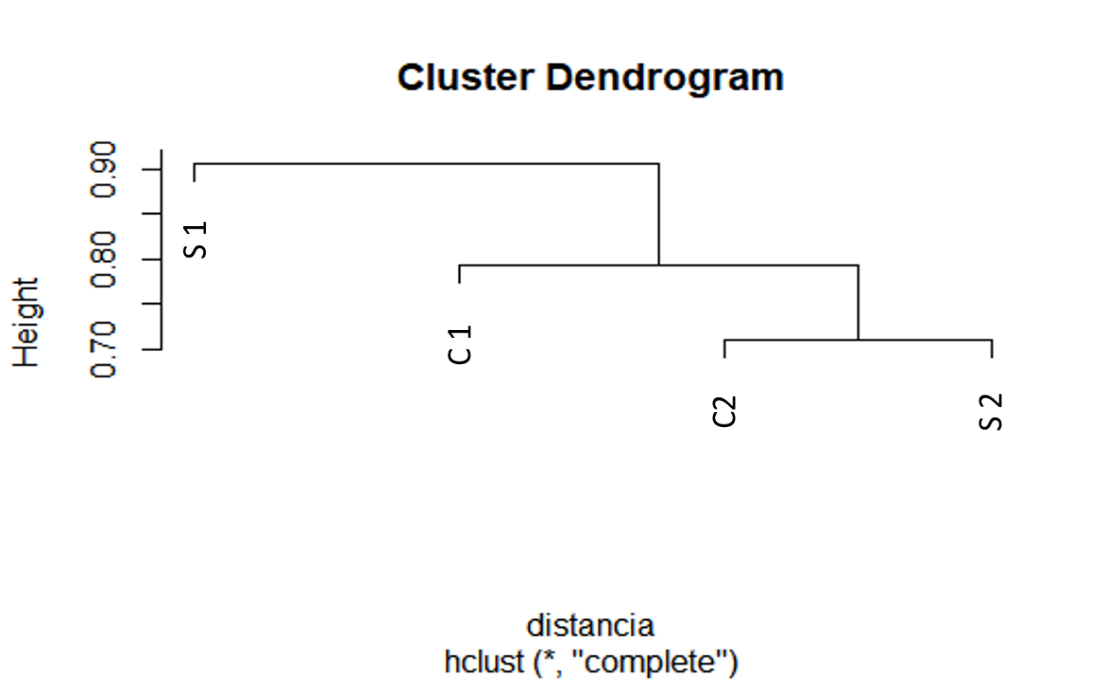

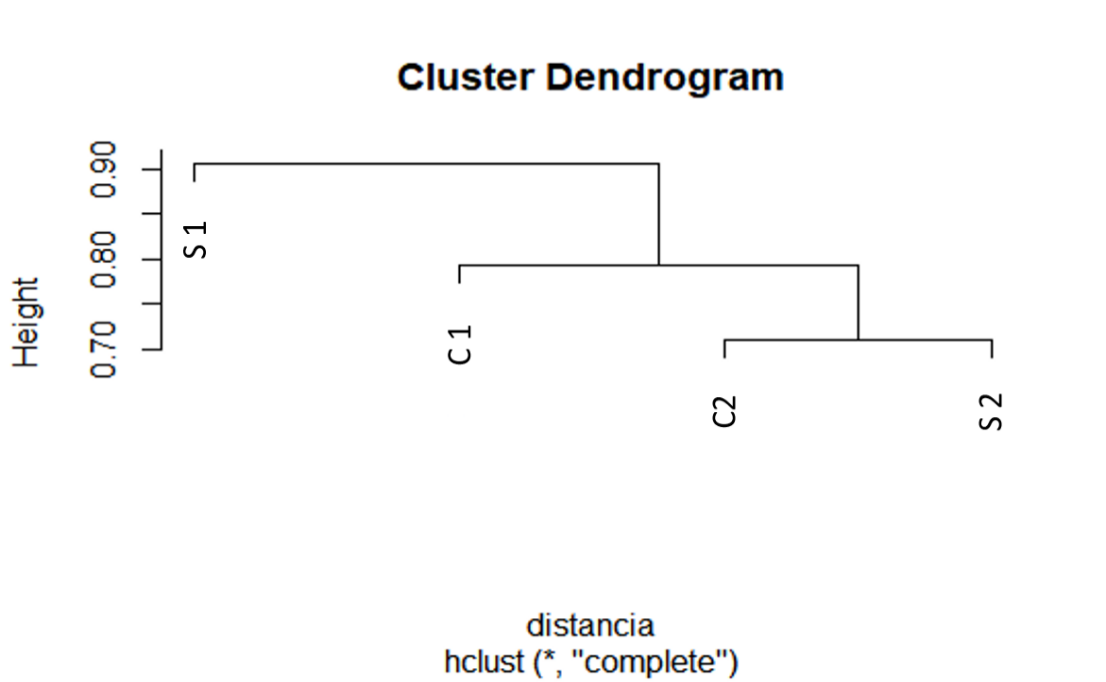

Supplement: S3 Fig — (DOCX) [file pone.0312624.s003.docx]
