# Supplementary material for: Fungi from Anopheles darlingi Root, 1926, larval breeding sites in the Brazilian Amazon
Source: PLoS One. 2024 Dec 5;19(12):e0312624. doi: 10.1371/journal.pone.0312624 (PMC11620424; doi:10.1371/journal.pone.0312624)
Supplement: S1 Table — (DOCX) [file pone.0312624.s004.docx]

**Supplementary Table 1.** Fungi isolated from *An. darlingi* breeding sites in the municipalities of Coari (C1 and C2) and São Gabriel da Cachoeira (S1 and S2).

| **LabMicra Code** | **GenBank deposit number** | **Taxon** | **Collection site** | **Isolation medium** | **Morphotype (Group)** |
| --- | --- | --- | --- | --- | --- |
| 1160 | MZ781268 | *Albifimbria* | C1 | SDAY | G0 |
| 1126 | MZ781261 | *Aspergillus* | C1 | ISP2 | G02 |
| 1148 | MZ781267 | *Aspergillus* | S1 | SDAY | G02 |
| 1171 |  | *Aspergillus* | C2 | PDA+L | G02 |
| 1204 |  | *Aspergillus* | C1 | AVA | G02 |
| 1209 |  | *Aspergillus* | C1 | SDAY | G02 |
| 1222 |  | *Aspergillus* | S2 | PDA+L | G02 |
| 1223 |  | *Aspergillus* | S2 | PDA+L | G02 |
| 1253 | MZ781287 | *Aspergillus* | S2 | SDAY | G02 |
| 1255 |  | *Aspergillus* | C1 | AVA | G02 |
| 1257 | MZ781289 | *Aspergillus* | S1 | AVA | G02 |
| 1259 |  | *Aspergillus* | C2 | PDA+L | G02 |
| 1283 | MZ781299 | *Aspergillus* | C1 | ISP2 | G02 |
| 1101 |  | *Chrysoporthe* | C1 | AVA | G04 |
| 1105 |  | *Chrysoporthe* | C2 | AVA | G04 |
| 1169 | MZ781272 | *Chrysoporthe* | C1 | PDA+L | G04 |
| 1213 |  | *Chrysoporthe* | S2 | ISP2 | G04 |
| 1132 | MZ781262 | *Cladosporium* | C1 | AVA | G05 |
| 1135 | MZ781264 | *Cladosporium* | C1 | PDA+L | G05 |
| 1078 | MZ781245 | Cucurbitariaceae | C2 | SDAY | G06 |
| 1196 |  | Cucurbitariaceae | C1 | AVA | G06 |
| 1093 | MZ781255 | *Cytospora* | C2 | SDAY | G07 |
| 1098 | MZ781256 | *Cytospora* | C2 | SDAY | G07 |
| 1106 | MZ781257 | *Cytospora* | C2 | SDAY | G07 |
| 1092 | MZ781254 | *Diaporthe* | C1 | SDAY | G08 |
| 1108 |  | *Diaporthe* | C2 | SDAY | G08 |
| 1194 |  | *Diaporthe* | S2 | AVA | G08 |
| 1200 |  | *Diaporthe* | C2 | AVA | G08 |
| 1203 | MZ781276 | *Diaporthe* | S2 | AVA | G08 |
| 1242 | MZ781281 | *Diaporthe* | S2 | PDA+L | G08 |
| 1231 |  | *Emmia* | S2 | SDAY | G03 |
| 1232 | MZ781279 | *Emmia* | S2 | SDAY | G03 |
| 1233 |  | *Emmia* | S2 | SDAY | G03 |
| 1236 |  | *Emmia* | S2 | PDA+L | G03 |
| 1248 | MZ781286 | *Epicoccum* | C1 | PDA+L | G09 |
| 1165 | MZ781271 | *Eutypella* | C2 | SDAY | G10 |
| 1240 | MZ781280 | *Eutypella* | C2 | SDAY | G10 |
| 1084 |  | *Fusarium* | C1 | SDAY | G11 |
| 1085 | MZ781250 | *Fusarium* | C1 | PDA+L | G11 |
| 1094 |  | *Fusarium* | C1 | SDAY | G11 |
| 1131 |  | *Fusarium* | C1 | SDAY | G11 |
| 1134 |  | *Fusarium* | C2 | PDA+L | G11 |
| 1143 |  | *Fusarium* | C1 | SDAY | G11 |
| 1144 |  | *Fusarium* | C1 | SDAY | G11 |
| 1146 |  | *Fusarium* | C1 | SDAY | G11 |
| 1149 |  | *Fusarium* | C1 | SDAY | G11 |
| 1154 |  | *Fusarium* | C1 | ISP2 | G11 |
| 1155 |  | *Fusarium* | C2 | ISP2 | G11 |
| 1182 |  | *Fusarium* | C2 | PDA+L | G11 |
| 1201 | MZ781275 | *Fusarium* | C2 | AVA | G11 |
| 1210 |  | *Fusarium* | C1 | SDAY | G11 |
| 1226 |  | *Fusarium* | S2 | SDAY | G11 |
| 1227 |  | *Fusarium* | S2 | ISP2 | G11 |
| 1262 | MZ781291 | *Fusarium* | C1 | SDAY | G11 |
| 1280 | MZ781298 | *Fusarium* | S2 | AVA | G11 |
| 1130 |  | *Fusarium* | C1 | SDAY | G11 |
| 1095 |  | *Gongronella* | C1 | AVA | G13 |
| 1111 | MZ781258 | *Gongronella* | C2 | PDA+L | G13 |
| 1207 |  | *Gongronella* | S2 | AVA | G13 |
| 1264 |  | *Gongronella* | S2 | ISP2 | G13 |
| 1267 |  | *Gongronella* | S2 | AVA | G13 |
| 1188 |  | *Hongkongmyces* | C2 | AVA | G14 |
| 1189 |  | *Hongkongmyces* | C2 | AVA | G14 |
| 1191 |  | *Hongkongmyces* | C2 | AVA | G14 |
| 1277 | MZ781297 | *Hongkongmyces* | C1 | PDA+L | G14 |
| 1158 |  | *Hongkongmyces* | C1 | AVA | G14 |
| 1157 |  | *Hyphodermella* | C1 | ISP2 | G26 |
| 1224 |  | *Hyphodermella.* | C2 | AVA | G26 |
| 1273 | MZ781296 | *Hyphodermella.* | S2 | ISP2 | G26 |
| 1167 |  | *Hypomontagnella* | C1 | AVA | G15 |
| 1197 |  | *Hypomontagnella* | C1 | PDA+L | G15 |
| 1199 |  | *Hypomontagnella* | C1 | AVA | G15 |
| 1205 | MZ781277 | *Hypomontagnella* | S2 | AVA | G15 |
| 1208 |  | *Hypomontagnella* | S2 | ISP2 | G15 |
| 1238 |  | *Hypomontagnella* | S2 | SDAY | G15 |
| 1239 |  | *Hypomontagnella* | S2 | SDAY | G15 |
| 1099 |  | *Microsphaeropsis* | C2 | AVA | G16 |
| 1082 | MZ781248 | *Microsphaeropsis* | C1 | SDAY | G16 |
| 1097 |  | *Microsphaeropsis* | C1 | PDA+L | G16 |
| 1102 |  | *Microsphaeropsis* | C1 | PDA+L | G16 |
| 1103 |  | *Microsphaeropsis* | C1 | PDA+L | G16 |
| 1147 |  | *Microsphaeropsis* | C2 | SDAY | G16 |
| 1151 |  | *Microsphaeropsis* | C2 | SDAY | G16 |
| 1162 |  | *Microsphaeropsis* | C1 | SDAY | G16 |
| 1168 |  | *Microsphaeropsis* | C1 | AVA | G16 |
| 1174 |  | *Microsphaeropsis* | C1 | ISP2 | G16 |
| 1175 |  | *Microsphaeropsis* | C1 | ISP2 | G16 |
| 1176 |  | *Microsphaeropsis* | C1 | ISP2 | G16 |
| 1178 |  | *Microsphaeropsis* | C1 | ISP2 | G16 |
| 1179 |  | *Microsphaeropsis* | C1 | ISP2 | G16 |
| 1183 |  | *Microsphaeropsis* | C1 | PDA+L | G16 |
| 1185 |  | *Microsphaeropsis* | C1 | PDA+L | G16 |
| 1186 |  | *Microsphaeropsis* | C1 | AVA | G16 |
| 1190 |  | *Microsphaeropsis* | C1 | SDAY | G16 |
| 1279 |  | *Microsphaeropsis* | C1 | ISP2 | G16 |
| 1079 | MZ781246 | *Nigrograna* | C1 | SDAY | G17 |
| 1122 |  | *Ochronis* | C1 | ISP2 | G01 |
| 1123 | MZ781259 | *Ochronis* | C1 | ISP2 | G01 |
| 1125 | MZ781260 | *Ochronis* | C2 | ISP2 | G01 |
| 1145 |  | *Ochronis* | C1 | SDAY | G01 |
| 1159 |  | *Ochronis* | C2 | ISP2 | G01 |
| 1161 |  | *Ochronis* | C2 | AVA | G01 |
| 1237 |  | *Ochronis* | C1 | PDA+L | G01 |
| 1081 |  | *Ochronis* | C1 | AVA | G01 |
| 1172 |  | *Ochronis* | C1 | ISP2 | G01 |
| 1216 |  | *Ochronis* | C1 | PDA+L | G01 |
| 1218 |  | *Ochronis* | C1 | PDA+L | G01 |
| 1229 |  | *Ochronis* | S2 | AVA | G01 |
| 1083 | MZ781249 | *Paraconiothyrium* | C1 | SDAY | G18 |
| 1142 |  | *Paraconiothyrium* | S1 | SDAY | G18 |
| 1163 |  | *Paraconiothyrium* | C1 | PDA+L | G18 |
| 1166 | MZ781270 | *Paraconiothyrium* | C1 | AVA | G18 |
| 1177 |  | *Paraconiothyrium* | C1 | ISP2 | G18 |
| 1180 |  | *Paraconiothyrium* | C1 | ISP2 | G18 |
| 1184 | MZ781274 | *Paraconiothyrium* | C1 | PDA+L | G18 |
| 1192 |  | *Paraconiothyrium* | C1 | PDA+L | G18 |
| 1193 |  | *Paraconiothyrium* | C1 | ISP2 | G18 |
| 1195 |  | *Paraconiothyrium* | C1 | ISP2 | G18 |
| 1225 |  | *Paraconiothyrium* | S1 | ISP2 | G18 |
| 1256 | MZ781288 | *Paraconiothyrium* | C1 | AVA | G18 |
| 1261 | MZ781290 | *Paraconiothyrium* | C1 | AVA | G18 |
| 1265 | MZ781293 | *Paraconiothyrium* | S2 | SDAY | G18 |
| 1275 |  | *Paraconiothyrium* | S1 | PDA+L | G18 |
| 1276 |  | *Paraconiothyrium* | C1 | ISP2 | G18 |
| 1080 | MZ781247 | *Paraconiothyrium* | C1 | AVA | G18 |
| 1088 |  | *Paraconiothyrium* | C1 | PDA+L | G18 |
| 1119 |  | *Paraconiothyrium* | C1 | ISP2 | G18 |
| 1124 |  | *Paraconiothyrium* | C1 | ISP2 | G18 |
| 1211 | MZ781278 | *Paraconiothyrium* | C1 | PDA+L | G18 |
| 1215 |  | *Paraconiothyrium* | C1 | ISP2 | G18 |
| 1217 |  | *Paraconiothyrium* | C1 | PDA+L | G18 |
| 1138 | MZ781266 | *Penicillium* | C1 | ISP2 | G19 |
| 1127 |  | *Penicillium* | C2 | PDA+L | G19 |
| 1212 |  | *Penicillium* | S2 | AVA | G19 |
| 1214 |  | *Penicillium* | S2 | ISP2 | G19 |
| 1219 |  | *Penicillium* | S1 | PDA+L | G19 |
| 1220 |  | *Penicillium* | S2 | ISP2 | G19 |
| 1235 |  | *Penicillium* | S2 | AVA | G19 |
| 1245 | MZ781283 | *Penicillium* | S2 | AVA | G19 |
| 1249 |  | *Penicillium* | S2 | ISP2 | G19 |
| 1250 |  | *Penicillium* | S2 | AVA | G19 |
| 1251 |  | *Penicillium* | C1 | SDAY | G19 |
| 1254 |  | *Penicillium* | S2 | AVA | G19 |
| 1260 |  | *Penicillium* | S2 | PDA+L | G19 |
| 1281 |  | *Penicillium* | S2 | AVA | G19 |
| 1107 |  | *Peniophora* | C1 | SDAY | G20 |
| 1109 |  | *Peniophora* | C1 | SDAY | G20 |
| 1113 |  | *Peniophora* | C1 | SDAY | G20 |
| 1150 |  | *Peniophora* | C1 | SDAY | G20 |
| 1164 | MZ781269 | *Peniophora* | C1 | SDAY | G20 |
| 1181 | MZ781273 | *Pyrenochaetopsis* | C1 | ISP2 | G21 |
| 1202 |  | *Pyrenochaetopsis* | C1 | AVA | G21 |
| 1272 | MZ781295 | *Pyrenochaetopsis* | C1 | PDA+L | G21 |
| 1278 |  | *Pyrenochaetopsis* | C1 | AVA | G21 |
| 1091 | MZ781253 | *Sarocladium* | C1 | SDAY | G22 |
| 1258 |  | *Sarocladium.* | S2 | SDAY | G22 |
| 1252 |  | *Sarocladium.* | S2 | PDA+L | G22 |
| 1266 | MZ781294 | *Sarocladium.* | S2 | PDA+L | G22 |
| 1269 |  | *Sarocladium.* | C2 | PDA+L | G22 |
| 1086 |  | *Striaticonidium* | C1 | PDA+L | G23 |
| 1089 | MZ781252 | *Striaticonidium* | C2 | PDA+L | G23 |
| 1274 |  | *Striaticonidium* | S2 | PDA+L | G23 |
| 1087 | MZ781251 | *Talaromyces* | C2 | PDA+L | G24 |
| 1096 |  | *Talaromyces* | C2 | AVA | G24 |
| 1118 |  | *Talaromyces* | C2 | AVA | G24 |
| 1120 |  | *Talaromyces* | C1 | ISP2 | G24 |
| 1198 |  | *Talaromyces* | C1 | SDAY | G24 |
| 1244 | MZ781282 | *Talaromyces* | C2 | PDA+L | G24 |
| 1246 | MZ781284 | *Talaromyces* | S2 | ISP2 | G24 |
| 1263 | MZ781292 | *Talaromyces* | S1 | PDA+L | G24 |
| 1100 |  | *Trametes* | C1 | SDAY | G12 |
| 1112 |  | *Trametes* | C2 | PDA+L | G12 |
| 1115 |  | *Trametes* | C1 | ISP2 | G12 |
| 1116 |  | *Trametes* | C2 | ISP2 | G12 |
| 1117 |  | *Trametes* | C1 | ISP2 | G12 |
| 1241 |  | *Trametes* | S2 | SDAY | G12 |
| 1243 |  | *Trametes* | S1 | AVA | G12 |
| 1247 | MZ781285 | *Trametes* | S2 | SDAY | G12 |
| 1104 |  | *Trichoderma* | C1 | PDA+L | G25 |
| 1110 |  | *Trichoderma* | C2 | PDA+L | G25 |
| 1114 |  | *Trichoderma* | C1 | ISP2 | G25 |
| 1121 |  | *Trichoderma* | C1 | ISP2 | G25 |
| 1133 | MZ781263 | *Trichoderma* | C2 | PDA+L | G25 |
| 1136 | MZ781265 | *Trichoderma* | C2 | PDA+L | G25 |
| 1137 |  | *Trichoderma* | C1 | PDA+L | G25 |
| 1139 |  | *Trichoderma* | C1 | ISP2 | G25 |
| 1140 |  | *Trichoderma* | C2 | ISP2 | G25 |
| 1141 |  | *Trichoderma* | C2 | ISP2 | G25 |
| 1187 |  | *Trichoderma* | C1 | AVA | G25 |
| 1206 |  | *Trichoderma* | S2 | AVA | G25 |
| 1221 |  | *Trichoderma* | S2 | ISP2 | G25 |
| 1228 |  | *Trichoderma* | S1 | SDAY | G25 |
| 1230 |  | *Trichoderma* | S2 | ISP2 | G25 |
| 1234 |  | *Trichoderma* | S2 | SDAY | G25 |
| 1268 |  | *Trichoderma* | C2 | AVA | G25 |
| 1153 |  | Fungo NID1 | C1 | ISP2 | G27 |
| 1156 |  | Fungo NID1 | C1 | ISP2 | G27 |
| 1170 |  | Fungo NID1 | C1 | PDA+L | G27 |
| 1173 |  | Fungo NID1 | C1 | ISP2 | G27 |
| 1270 |  | Fungo NID1 | C1 | SDAY | G27 |
| 1271 |  | Fungo NID1 | C1 | SDAY | G27 |
| 1282 |  | Fungo NID2 | C2 | PDA+L | G28 |
| 1090 |  | Fungo NID3 | C2 | SDAY | G29 |
| 1128 |  | Fungo NID3 | C2 | PDA+L | G29 |
| 1129 |  | Fungo NID3 | C1 | SDAY | G29 |
| 1152 |  | Fungo NID3 | S1 | SDAY | G29 |

Isolated and preserved strains were deposited in the LaBMicrA/UFAM work collection (LabMicrA code) and registered under the SisGen (National System for the Management of Genetic Heritage and Traditional Knowledge Associated) number AD64E07. rDNA sequences generated for selected strains were deposited in the NCBI GenBank database (accession numbers MZ781245 - MZ781299). Using phylogenetic analysis (Supplementary Figure 3). Sequences recovered from this study that were placed in taxon-specific bootstrap-supported monophyletic groups with GenBank reference sequences were assigned to taxa they grouped with. Isolation Culture media: AVA (oats, agar, dextrose, yeast extract, and malt extract), PDA + L (potato, dextrose, and agar plus yeast extract), ISP2 (agar, starch, dextrose, yeast extract, and extract malt), and SDAY (agar, dextrose, yeast extract, and peptone).
