# Supplementary material for: Fungi from Anopheles darlingi Root, 1926, larval breeding sites in the Brazilian Amazon
Source: PLoS One. 2024 Dec 5;19(12):e0312624. doi: 10.1371/journal.pone.0312624 (PMC11620424; doi:10.1371/journal.pone.0312624)
Supplement: S2 Table — (DOCX) [file pone.0312624.s005.docx]

**Supplementary Table 2.** Fungi isolates from *An. darlingi* larval breeding sites located in the municipalities of Coari (C1 and C2) and São Gabriel da Cachoeira (S1 and S2).

| **Collection site** | **Isolated fungi** | **Morphotypes (similar morphological characteristics)** | **High quality rDNA ITS sequences^2^** |
| --- | --- | --- | --- |
| C1 | 107 (52%) | 26 (86.6%) | 26 (24.3%) |
| C2 | 44 (21%) | 20 (66.6%) | 14 (31.8%) |
| S1 | 10 (5%) | 7 (23.3%) | 3 (30.0%) |
| S2 | 45 (22%) | 16 (53.3%) | 12 (26.7%) |
| Total | 206 (100%) | 30^1^ | 55 (26.7%) |

^1^Total of morphological groups obtained in this work. ^2^Values in brackets are percentages from total analyzed fungi.
