# Supplementary material for: Fungi from Anopheles darlingi Root, 1926, larval breeding sites in the Brazilian Amazon
Source: PLoS One. 2024 Dec 5;19(12):e0312624. doi: 10.1371/journal.pone.0312624 (PMC11620424; doi:10.1371/journal.pone.0312624)
Supplement: S3 Table — (DOCX) [file pone.0312624.s006.docx]

**Supplementary Table 3.** Taxonomic classification of fungi isolated from *An. darlingi* breeding sites in the municipalities of Coari (C1 and C2) and São Gabriel da Cachoeira (S1 and S2), based on sequencing of the nuclear ribosomal internal transcribed spacer (ITS) region and comparison of the sequences with those in the NCBI database, and analysis of the phylogenetic trees.

| **LabMicra Code** | **GenBank Deposit number** | | **LabMicra Code** | **Taxonomic Classification** | | **% Identity** | **Reference ID in GenBank** | **GenBank Accession number** | |
| --- | --- | --- | --- | --- | --- | --- | --- | --- | --- |
| 1078 | M01 | | MZ781245 | Cucurbitariaceae | | 99.79% | [Cucurbitariaceae](https://blast.ncbi.nlm.nih.gov/Blast.cgi#alnHdr_1285276478) | [LT592923.1](https://www.ncbi.nlm.nih.gov/nucleotide/LT592923.1?report=genbank&log%24=nucltop&blast_rank=1&RID=G4KRD752013) | |
| 1160 | M83 | | MZ781268 | *Albifimbria lateralis* | | 100.00% | [*Al. lateralis*](https://blast.ncbi.nlm.nih.gov/Blast.cgi#alnHdr_1339503319) | [NR_153548.1](https://www.ncbi.nlm.nih.gov/nucleotide/NR_153548.1?report=genbank&log%24=nucltop&blast_rank=2&RID=G4VG0MTV013) | |
| 1148 | M71 | | MZ781267 | *Aspergillus hortai* | | 100.00% | [*A. hortai*](https://blast.ncbi.nlm.nih.gov/Blast.cgi#alnHdr_985525865) | [KP987087.1](https://www.ncbi.nlm.nih.gov/nucleotide/KP987087.1?report=genbank&log%24=nucltop&blast_rank=1&RID=G4VA36JF016) | |
| 1257 | M179 | | MZ781289 | *Aspergillus hortai* | | 100.00% | [*A. hortai*](https://blast.ncbi.nlm.nih.gov/Blast.cgi#alnHdr_985525865) | [KP987087.1](https://www.ncbi.nlm.nih.gov/nucleotide/KP987087.1?report=genbank&log%24=nucltop&blast_rank=1&RID=G4VA36JF016) | |
| 1283 | M205 | | MZ781299 | *Aspergillus hortai* | | 100.00% | [*A.s hortai*](https://blast.ncbi.nlm.nih.gov/Blast.cgi#alnHdr_985525865) | [KP987087.1](https://www.ncbi.nlm.nih.gov/nucleotide/KP987087.1?report=genbank&log%24=nucltop&blast_rank=1&RID=G4VA36JF016) | |
| 1126 | M49 | | MZ781261 | *Aspergillus* sp. nov. | | 95.65% | [*A. iranicus*](https://blast.ncbi.nlm.nih.gov/Blast.cgi#alnHdr_1385000022) | [NR_156297.1](https://www.ncbi.nlm.nih.gov/nucleotide/NR_156297.1?report=genbank&log%24=nucltop&blast_rank=16&RID=G4TX8M76016) | |
| 1253 | M175 | | MZ781287 | *Aspergillus* sp. nov. | | 94.60% | *A. homomorphus* | [MH862768.1](https://www.ncbi.nlm.nih.gov/nucleotide/MH862768.1?report=genbank&log%24=nucltop&blast_rank=21&RID=GCNPF1E1013) | |
| 1169 | M91 | | MZ781272 | *Chrysoporther* sp. | | 98.39% | [*Ch. hodgesiana*](https://blast.ncbi.nlm.nih.gov/Blast.cgi#alnHdr_358001425) | [JN942329.1](https://www.ncbi.nlm.nih.gov/nucleotide/JN942329.1?report=genbank&log%24=nucltop&blast_rank=14&RID=G4WAGHBT013) | |
| 1132 | M55 | | MZ781262 | *Cladosporium* sp. | | 98.24% | [*Cl. ramotenellum*](https://blast.ncbi.nlm.nih.gov/Blast.cgi#alnHdr_1473250183) | [MH863127.1](https://www.ncbi.nlm.nih.gov/nucleotide/MH863127.1?report=genbank&log%24=nucltop&blast_rank=61&RID=G4U92S0T013) | |
| 1135 | M58 | | MZ781264 | *Cladosporium* sp. | | 98.24% | [*Cl. ramotenellum*](https://blast.ncbi.nlm.nih.gov/Blast.cgi#alnHdr_1473250183) | [MH863127.1](https://www.ncbi.nlm.nih.gov/nucleotide/MH863127.1?report=genbank&log%24=nucltop&blast_rank=61&RID=G4U92S0T013) | |
| 1093 | M16 | | MZ781255 | *Cytospora* sp. nov. | | 97.21% | [*C. lumnitzericola*](https://blast.ncbi.nlm.nih.gov/Blast.cgi#alnHdr_1346797852) | [MG975778.1](https://www.ncbi.nlm.nih.gov/nucleotide/MG975778.1?report=genbank&log%24=nucltop&blast_rank=4&RID=G4ST0N3G013) | |
| 1098 | M21 | | MZ781256 | *Cytospora* sp. nov. | | 97.11% | [*C.lumnitzericola*](https://blast.ncbi.nlm.nih.gov/Blast.cgi#alnHdr_2044083742) | [NR_172402.1](https://www.ncbi.nlm.nih.gov/nucleotide/NR_172402.1?report=genbank&log%24=nucltop&blast_rank=6&RID=G4SY5DNB013) | |
| 1106 | M29 | | MZ781257 | *Cytospora s*p. nov. | | 97.23% | [*C. lumnitzericola*](https://blast.ncbi.nlm.nih.gov/Blast.cgi#alnHdr_2044083742) | [NR_172402.1](https://www.ncbi.nlm.nih.gov/nucleotide/NR_172402.1?report=genbank&log%24=nucltop&blast_rank=6&RID=G4TGN6RW016) | |
| 1092 | M15 | | MZ781254 | *Diaporthe* sp. nov. | | 98.98% | [*D. ueckerae*](https://blast.ncbi.nlm.nih.gov/Blast.cgi#alnHdr_1194526854) | [NR_147543.1](https://www.ncbi.nlm.nih.gov/nucleotide/NR_147543.1?report=genbank&log%24=nucltop&blast_rank=5&RID=G5Y27GE6016) | |
| 1203 | M125 | | MZ781276 | *Diaporther ueckerae* | | 99.00% | [*D. ueckerae*](https://blast.ncbi.nlm.nih.gov/Blast.cgi#alnHdr_1194526854) | [NR_147543.1](https://www.ncbi.nlm.nih.gov/nucleotide/NR_147543.1?report=genbank&log%24=nucltop&blast_rank=5&RID=GCMBY4YD013) | |
| 1242 | M164 | | MZ781281 | *Diaporther ueckerae* | | 99.00% | [*D. ueckerae*](https://blast.ncbi.nlm.nih.gov/Blast.cgi#alnHdr_1194526854) | [NR_147543.1](https://www.ncbi.nlm.nih.gov/nucleotide/NR_147543.1?report=genbank&log%24=nucltop&blast_rank=5&RID=GCMBY4YD013) | |
| 1232 | M154 | | MZ781279 | *Emmia* sp. nov. | | 98.46% | [*Em. latemarginata*](https://blast.ncbi.nlm.nih.gov/Blast.cgi#alnHdr_1473243483) | [MH856427.1](https://www.ncbi.nlm.nih.gov/nucleotide/MH856427.1?report=genbank&log%24=nucltop&blast_rank=83&RID=G8RTYDF001R) | |
| 1248 | M170 | | MZ781286 | *Epicoccum latusicollum* | | 100.00% | [*Ep. latusicollum*](https://blast.ncbi.nlm.nih.gov/Blast.cgi#alnHdr_1216872508) | [KY742101.1](https://www.ncbi.nlm.nih.gov/nucleotide/KY742101.1?report=genbank&log%24=nucltop&blast_rank=1&RID=G5XK9SMN016) | |
| 1166 | M88 | | MZ781270 | *Eutypella scoparia* | | 98.31% | *E. scoparia* | [NR_166007.1](https://www.ncbi.nlm.nih.gov/nucleotide/NR_166007.1?report=genbank&log%24=nucltop&blast_rank=1&RID=G4VWJBYG016) | |
| 1240 | M162 | | MZ781280 | *Eutypella* sp. nov*.* | | 85.89% | [*E. parasitica*](https://blast.ncbi.nlm.nih.gov/Blast.cgi#alnHdr_1818327614) | [NR_165995.1](https://www.ncbi.nlm.nih.gov/nucleotide/NR_165995.1?report=genbank&log%24=nucltop&blast_rank=11&RID=G8RA88H3016) | |
| 1262 | M184 | MZ781291 | | *Fusarium oxysporum* | 100.00% | | [*F. oxysporum*](https://blast.ncbi.nlm.nih.gov/Blast.cgi#alnHdr_1783302001) | | [MN817703.1](https://www.ncbi.nlm.nih.gov/nucleotide/MN817703.1?report=genbank&log%24=nucltop&blast_rank=9&RID=G8P8RRUH016) |
| 1280 | M202 | MZ781298 | | *Fusarium oxysporum* | 100.00% | | [*F. oxysporum*](https://blast.ncbi.nlm.nih.gov/Blast.cgi#alnHdr_1783302001) | | [MN817703.1](https://www.ncbi.nlm.nih.gov/nucleotide/MN817703.1?report=genbank&log%24=nucltop&blast_rank=9&RID=G8P8RRUH016) |
| 1085 | M08 | MZ781250 | | *Fusarium* sp. | 99.58% | | [*F. spathulatum*](https://blast.ncbi.nlm.nih.gov/Blast.cgi#alnHdr_168419825) | | [EU329674.1](https://www.ncbi.nlm.nih.gov/nucleotide/EU329674.1?report=genbank&log%24=nucltop&blast_rank=2&RID=G8NFYU74016) |
| 1201 | M123 | MZ781275 | | *Fusarium* sp. | 99.58% | | [*F. spathulatum*](https://blast.ncbi.nlm.nih.gov/Blast.cgi#alnHdr_168419825) | | [EU329674.1](https://www.ncbi.nlm.nih.gov/nucleotide/EU329674.1?report=genbank&log%24=nucltop&blast_rank=2&RID=G8NFYU74016) |
| 1111 | M34 | MZ781258 | | *Gongronella butleri* | 99.80% | | *G. butleri* | | [JN942999.1](https://www.ncbi.nlm.nih.gov/nucleotide/JN942999.1?report=genbank&log%24=nucltop&blast_rank=20&RID=GCKG7THK013) |
| 1277 | M199 | MZ781297 | | *Hongkongmyces* sp. nov | 96.09% | | *H. pedis* | | [NR_149338.1](https://www.ncbi.nlm.nih.gov/nucleotide/NR_149338.1?report=genbank&log%24=nucltop&blast_rank=1&RID=GCN0BZ0Y013) |
| 1273 | M195 | MZ781296 | | *Hyphodermella* sp. nov*.* | 92.12% | | [*Hy. sp.*](https://blast.ncbi.nlm.nih.gov/Blast.cgi#alnHdr_1026666427) | | [KX008367.1](https://www.ncbi.nlm.nih.gov/nucleotide/KX008367.1?report=genbank&log%24=nucltop&blast_rank=44&RID=G5WF4YUR013) |
| 1205 | M127 | MZ781277 | | *Hypomontagnella*  *monticulosa* | 99.56% | | [*Hy. monticulosa*](https://blast.ncbi.nlm.nih.gov/Blast.cgi#alnHdr_1510262302) | | [MK131719.1](https://www.ncbi.nlm.nih.gov/nucleotide/MK131719.1?report=genbank&log%24=nucltop&blast_rank=41&RID=G8S7T0N2016) |
| 1082 | M05 | MZ781248 | | *Microsphaeropsis arundinis* | 99.27% | | [*M. arundinis*](https://blast.ncbi.nlm.nih.gov/Blast.cgi#alnHdr_595827215) | | [JX496010.1](https://www.ncbi.nlm.nih.gov/nucleotide/JX496010.1?report=genbank&log%24=nucltop&blast_rank=2&RID=G4NGV3XR016) |
| 1079 | M02 | MZ781246 | | *Nigrograna chromolaenae* | 98.92% | | [*N. mackinnonii*](https://blast.ncbi.nlm.nih.gov/Blast.cgi#alnHdr_825706259) | | [NR_132037.1](https://www.ncbi.nlm.nih.gov/nucleotide/NR_132037.1?report=genbank&log%24=nucltop&blast_rank=7&RID=G4SEM80V013) |
| 1123 | M46 | MZ781259 | | *Ochroconis* sp.nov. | 90.70% | | [*O. minima*](https://blast.ncbi.nlm.nih.gov/Blast.cgi#alnHdr_1126634308) | | [NR_145366.1](https://www.ncbi.nlm.nih.gov/nucleotide/NR_145366.1?report=genbank&log%24=nucltop&blast_rank=13&RID=G4TRFTXV016) |
| 1125 | M48 | MZ781260 | | *Ochroconis* sp.nov. | 90.70% | | [*O. minima*](https://blast.ncbi.nlm.nih.gov/Blast.cgi#alnHdr_1126634308) | | [NR_145366.1](https://www.ncbi.nlm.nih.gov/nucleotide/NR_145366.1?report=genbank&log%24=nucltop&blast_rank=13&RID=G4TRFTXV016) |
| 1165 | M88e | MZ781271 | | *Paraconiothyrium cyclothyrioides* | 99.62% | | *P. cyclothyrioides* | | [LT796894.1](https://www.ncbi.nlm.nih.gov/nucleotide/LT796894.1?report=genbank&log%24=nucltop&blast_rank=5&RID=GCKW9KZR013) |
| 1083 | M06 | MZ781249 | | *Paraconiothyrium estuarium* | 99.25% | | [*Pa. estuarinum*](https://blast.ncbi.nlm.nih.gov/Blast.cgi#alnHdr_1818327629) | | NR_166007.1 |
| 1184 | M106 | MZ781274 | | *Paraconiothyrium estuarium* | 98.31% | | [*Pa. estuarinum*](https://blast.ncbi.nlm.nih.gov/Blast.cgi#alnHdr_1818327629) | | [NR_166007.1](https://www.ncbi.nlm.nih.gov/nucleotide/NR_166007.1?report=genbank&log%24=nucltop&blast_rank=1&RID=G4VWJBYG016) |
| 1256 | M178 | MZ781288 | | *Paraconiothyrium estuarium* | 98.31% | | [*Pa. estuarinum*](https://blast.ncbi.nlm.nih.gov/Blast.cgi#alnHdr_1818327629) | | [NR_166007.1](https://www.ncbi.nlm.nih.gov/nucleotide/NR_166007.1?report=genbank&log%24=nucltop&blast_rank=1&RID=G4VWJBYG016) |
| 1261 | M183e | MZ781290 | | *Paraconiothyrium estuarium* | 98.31% | | [*Pa. estuarinum*](https://blast.ncbi.nlm.nih.gov/Blast.cgi#alnHdr_1818327629) | | [NR_166007.1](https://www.ncbi.nlm.nih.gov/nucleotide/NR_166007.1?report=genbank&log%24=nucltop&blast_rank=1&RID=G4VWJBYG016) |
| 1265 | M187 | MZ781293 | | *Paraconiothyrium estuarium* | 99.64% | | [*Pa. estuarinum*](https://blast.ncbi.nlm.nih.gov/Blast.cgi#alnHdr_1818327629) | | [NR_166007.1](https://www.ncbi.nlm.nih.gov/nucleotide/NR_166007.1?report=genbank&log%24=nucltop&blast_rank=1&RID=G4VWJBYG016) |
| 1080 | M03 | MZ781247 | | *Paraconiothyrium* sp. nov*.* | 99.25% | | [*Pa. salinum*](https://blast.ncbi.nlm.nih.gov/Blast.cgi#alnHdr_1729918339) | | [MN369540.1](https://www.ncbi.nlm.nih.gov/nucleotide/MN369540.1?report=genbank&log%24=nucltop&blast_rank=3&RID=G4MVRCVA01R) |
| 1211 | M133 | MZ781278 | | *Paraconiothyrium* sp. nov*.* | 99.27% | | [*Pa. salinum*](https://blast.ncbi.nlm.nih.gov/Blast.cgi#alnHdr_1729918339) | | [MN369540.1](https://www.ncbi.nlm.nih.gov/nucleotide/MN369540.1?report=genbank&log%24=nucltop&blast_rank=3&RID=G5YT25V2013) |
| 1138 | M61 | MZ781266 | | *Penicillium citrosulfuratum* | 100.00% | | [*Pe. citreosulfuratum*](https://blast.ncbi.nlm.nih.gov/Blast.cgi#alnHdr_820944598) | | [KP016814.1](https://www.ncbi.nlm.nih.gov/nucleotide/KP016814.1?report=genbank&log%24=nucltop&blast_rank=2&RID=G4V3H2SR016) |
| 1245 | M167 | MZ781283 | | *Penicillium citrosulfuratum* | 100.00% | | [*Pe. citreosulfuratum*](https://blast.ncbi.nlm.nih.gov/Blast.cgi#alnHdr_820944598) | | [KP016814.1](https://www.ncbi.nlm.nih.gov/nucleotide/KP016814.1?report=genbank&log%24=nucltop&blast_rank=2&RID=G5XY7BRX013) |
| 1164 | M87 | MZ781269 | | *Peniophora* sp. nov. | 98.87% | | [*P. guadelupensis*](https://blast.ncbi.nlm.nih.gov/Blast.cgi#alnHdr_1473249360) | | [MH862304.1](https://www.ncbi.nlm.nih.gov/nucleotide/MH862304.1?report=genbank&log%24=nucltop&blast_rank=19&RID=G8JW4FUC01R) |
| 1181 | M103 | MZ781273 | | *Pyrenochaetopsis sinensis* | 99.77% | | [*Py. sinensis*](https://blast.ncbi.nlm.nih.gov/Blast.cgi#alnHdr_1990716024) | | [NR_171834.1](https://www.ncbi.nlm.nih.gov/nucleotide/NR_171834.1?report=genbank&log%24=nucltop&blast_rank=15&RID=G8M095A6016) |
| 1272 | M194 | MZ781295 | | *Pyrenochaetopsis* sp. nov*.* | 98.21% | | *Py. poae* | | [KJ869117.1](https://www.ncbi.nlm.nih.gov/nucleotide/KJ869117.1?report=genbank&log%24=nucltop&blast_rank=7&RID=GCMTY98A013) |
| 1091 | M14 | MZ781253 | | *Sarocladium* sp. nov. | 93.99% | | [*S. oryzae*](https://blast.ncbi.nlm.nih.gov/Blast.cgi#alnHdr_1124130978) | | [NR_14504](https://www.ncbi.nlm.nih.gov/nucleotide/NR_145045.1?report=genbank&log%24=nucltop&blast_rank=6&RID=G4S89YX0016) |
| 1266 | M188 | MZ781294 | | *Sarocladium* sp. nov. | 92.45% | | [*S. gamsii*](https://blast.ncbi.nlm.nih.gov/Blast.cgi#alnHdr_1379127721) | | [NR_155780.1](https://www.ncbi.nlm.nih.gov/nucleotide/NR_155780.1?report=genbank&log%24=nucltop&blast_rank=1&RID=G5Z85X0R013) |
| 1089 | M12 | MZ781252 | | *Striaticonidium synnematum* | 98.57% | | [*St. synnematum*](https://blast.ncbi.nlm.nih.gov/Blast.cgi#alnHdr_1351337501) | | [NR_154432.1](https://www.ncbi.nlm.nih.gov/nucleotide/NR_154432.1?report=genbank&log%24=nucltop&blast_rank=1&RID=G4R0GFPX016) |
| 1263 | M185 | MZ781292 | | *Talaromyces amestolkiae* | 99.82% | | [*Ta. amestolkiae*](https://blast.ncbi.nlm.nih.gov/Blast.cgi#alnHdr_626617340) | | [NR_120179.1](https://www.ncbi.nlm.nih.gov/nucleotide/NR_120179.1?report=genbank&log%24=nucltop&blast_rank=1&RID=G5WVKA7R016) |
| 1087 | M10 | MZ781251 | | *Talaromyces* sp. nov | 96.72% | | [*Ta. cinnabarinus*](https://blast.ncbi.nlm.nih.gov/Blast.cgi#alnHdr_1473247527) | | [MH860471.1](https://www.ncbi.nlm.nih.gov/nucleotide/MH860471.1?report=genbank&log%24=nucltop&blast_rank=1&RID=G4PSGVTK01R) |
| 1244 | M166 | MZ781282 | | *Talaromyces* sp. nov | 96.67% | | [*Ta. cinnabarinus*](https://blast.ncbi.nlm.nih.gov/Blast.cgi#alnHdr_1473247527) | | [MH860471.1](https://www.ncbi.nlm.nih.gov/nucleotide/MH860471.1?report=genbank&log%24=nucltop&blast_rank=2&RID=G5X6BAFU013) |
| 1246 | M168 | MZ781284 | | *Talaromyces* sp. nov | 97.68% | | [*T. palmae*](https://blast.ncbi.nlm.nih.gov/Blast.cgi#alnHdr_511801442) | | [NR_103617.1](https://www.ncbi.nlm.nih.gov/nucleotide/NR_103617.1?report=genbank&log%24=nucltop&blast_rank=1&RID=G5WYB3G3013) |
| 1247 | M169 | MZ781285 | | *Trametes menziesii* | 99.58% | | [*Tr. menziesii*](https://blast.ncbi.nlm.nih.gov/Blast.cgi#alnHdr_530340216) | | [KC848326.1](https://www.ncbi.nlm.nih.gov/nucleotide/KC848326.1?report=genbank&log%24=nucltop&blast_rank=20&RID=G5XEJZKZ013) |
| 1133 | M56 | MZ781263 | | *Trichoderma atroviride* | 100.00% | | [*T. atroviride*](https://blast.ncbi.nlm.nih.gov/Blast.cgi#alnHdr_1718371448) | | [MN262486.1](https://www.ncbi.nlm.nih.gov/nucleotide/MN262486.1?report=genbank&log%24=nucltop&blast_rank=1&RID=G4UV66VC01R) |
| 1136 | M59 | MZ781265 | | *Trichoderma atroviride* | 100.00% | | [*T. atroviride*](https://blast.ncbi.nlm.nih.gov/Blast.cgi#alnHdr_1718371448) | | [MN262486.1](https://www.ncbi.nlm.nih.gov/nucleotide/MN262486.1?report=genbank&log%24=nucltop&blast_rank=1&RID=G4UV66VC01R) |
