# Supplementary material for: Fungi from Anopheles darlingi Root, 1926, larval breeding sites in the Brazilian Amazon
Source: PLoS One. 2024 Dec 5;19(12):e0312624. doi: 10.1371/journal.pone.0312624 (PMC11620424; doi:10.1371/journal.pone.0312624)
Supplement: S4 Table — (DOCX) [file pone.0312624.s007.docx]

**Supplementary Table 4.** Diversity estimates found at different collection sites.

| **Diversity index** | **Collection sites** | | | |
| --- | --- | --- | --- | --- |
|  | C1 | C2 | S1 | S2 |
| Taxa_S | 26 | 20 | 7 | 16 |
| Individuals_n | 107 | 44 | 10 | 45 |
| Simpson_1-D | 0.911 | 0.931 | 0.82 | 0.897 |
| Shannon_H’ | 2.772 | 2.831 | 1.834 | 2.52 |
| Equitability_J | 0.851 | 0.944 | 0.942 | 0.909 |
| Chao-1 | **33.5** | **24.67** | **12** | **21** |
