# Supplementary material for: Fungi from Anopheles darlingi Root, 1926, larval breeding sites in the Brazilian Amazon
Source: PLoS One. 2024 Dec 5;19(12):e0312624. doi: 10.1371/journal.pone.0312624 (PMC11620424; doi:10.1371/journal.pone.0312624)
Supplement: S5 Table — (DOCX) [file pone.0312624.s008.docx]

**Supplementary Table 5.** Kruskal-Wallis test with Dunn's post hoc tests to verify the difference in richness among the collection sites.

| **Comparison** | **Z** | **P.unadj** | **P.adj** | **p-value** |
| --- | --- | --- | --- | --- |
| **Collection sites** |  |  |  |  |
| C 1 - C2 | 2.3894612 | 1.687311e-02 | 2.024773e-02 | 0.002 |
| C1 - S1 | 5.4823062 | 4.198167e-08 | 2.518900e-07 | 0.00000002 |
| C2 - S1 | 3.0928450 | 1.982476e-03 | 5.947429e-03 | 0.0005 |
| C1 - S2 | 2.9123155 | 3.587600e-03 | 7.175201e-03 | 0.0007 |
| C2 - S2 | 0.5228543 | 6.010756e-01 | 6.010756e-01 | 0.06 |
| S1 - S2 | -2.5699907 | 1.017012e-02 | 1.525519e-02 | 0.001 |
